# Supplementary material for: Circadian clock regulator Bmal1 gates axon regeneration via Tet3 epigenetics in mouse sensory neurons
Source: Nat Commun. 2023 Aug 24;14:5165. doi: 10.1038/s41467-023-40816-7 (PMC10449865; doi:10.1038/s41467-023-40816-7)

SUPPLEMENTARY FIGURES

Halawani et al.

Circadian clock regulator Bmal1 gates axon regeneration via Tet3 epigenetics in mouse sensory neurons

Supplementary Figures S1-S17.

On following pages (including uncropped Western blots of supplementary figures).

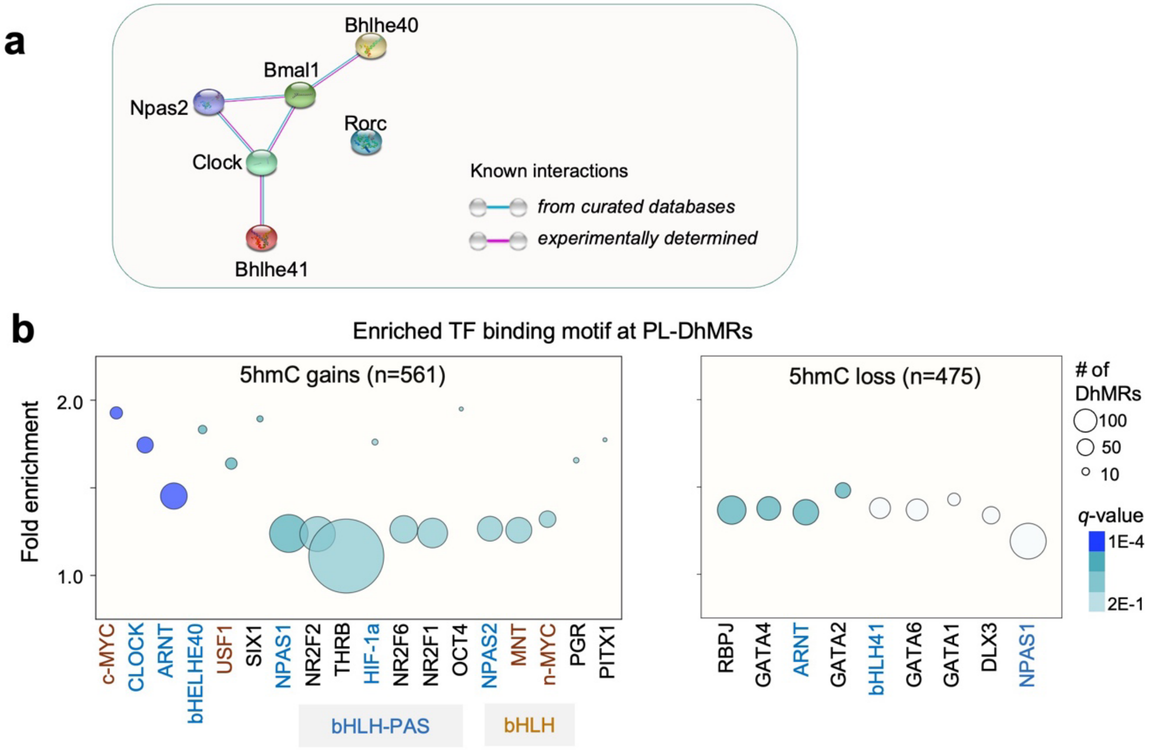

c

bHLH-PAS Regeneration Associated Genes

Gene	Reference
Bmal1	RAG: Blesch 2012; Palmisano 2019; Renthal 2020; this study
Clock	RAG: Renthal 2020; this study
Npas2	not RAG
Bhlhe40	RAG: Hu 2016; Chandran 2016; Renthal 2020
Bhlhe41	RAG: Hu 2016; Palmisano 2019; Renthal 2020
Rorc	not RAG
Hif1a	RAG: Blesch 2012; Chandran 2016; Geeven 2011; Renthal 2020

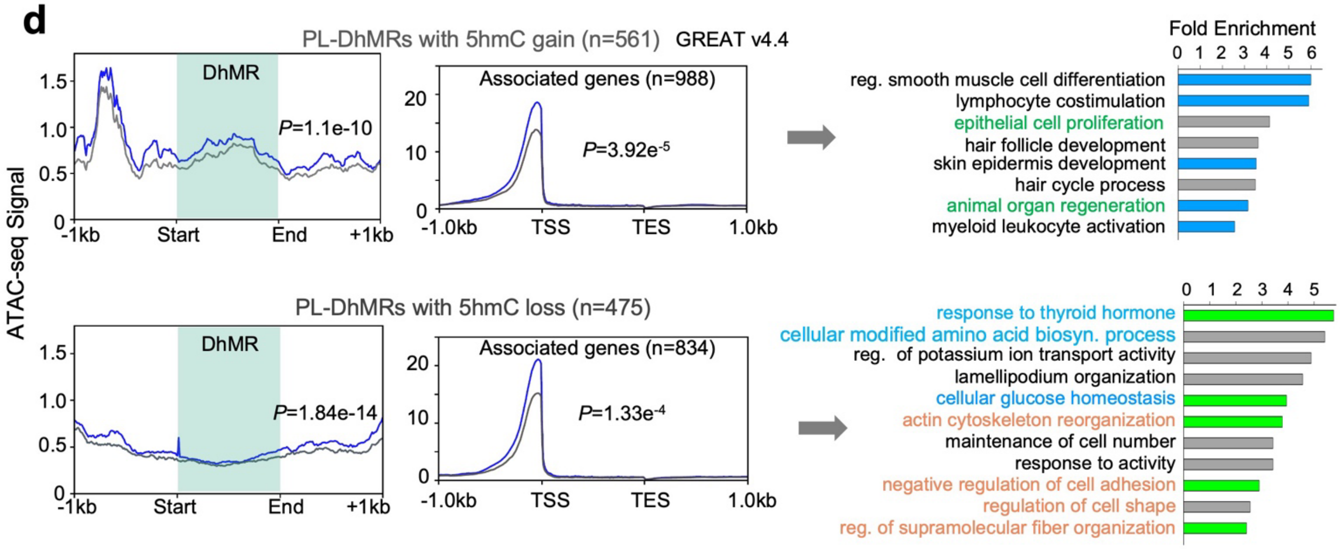

Figure S1. Enrichment of bHLH-PAS TFs in DhMRs displaying enhanced chromatin accessibility after peripheral lesion of DRG.

a. STRING protein association network analysis of circadian transcription factors whose binding motifs were found to be enriched in PL-DhMRs. Analysis was performed using default settings of medium confidence (0.4).

b. Bubble plots show enriched TF binding motifs in PL-specific DhMRs with 5hmC gain (n=561) or loss (n=475) analyzed separately. Members of the bHLH-PAS family are labeled in blue and members of the bHLH family in brown. Circle size denotes DhMR numbers and color scale significance of enrichment.

c. Tabular summary showing that most members of the bHLH-PAS transcription factors with enriched binding motifs in PL-DhMRs have been previously identified as regeneration associated genes (RAGs).

d. Left, analysis of ATAC-seq signals from DRGs after sham or PL at 1 dpi (Palmisano et al., 2019) shows increased chromatin accessibility at PL-specific DhMRs, more so for those with 5hmC gain. TSS, transcription start site; TES, transcription end site. Welch's two-sample t-test. Right, enriched GO terms of biological processes for the associated genes.

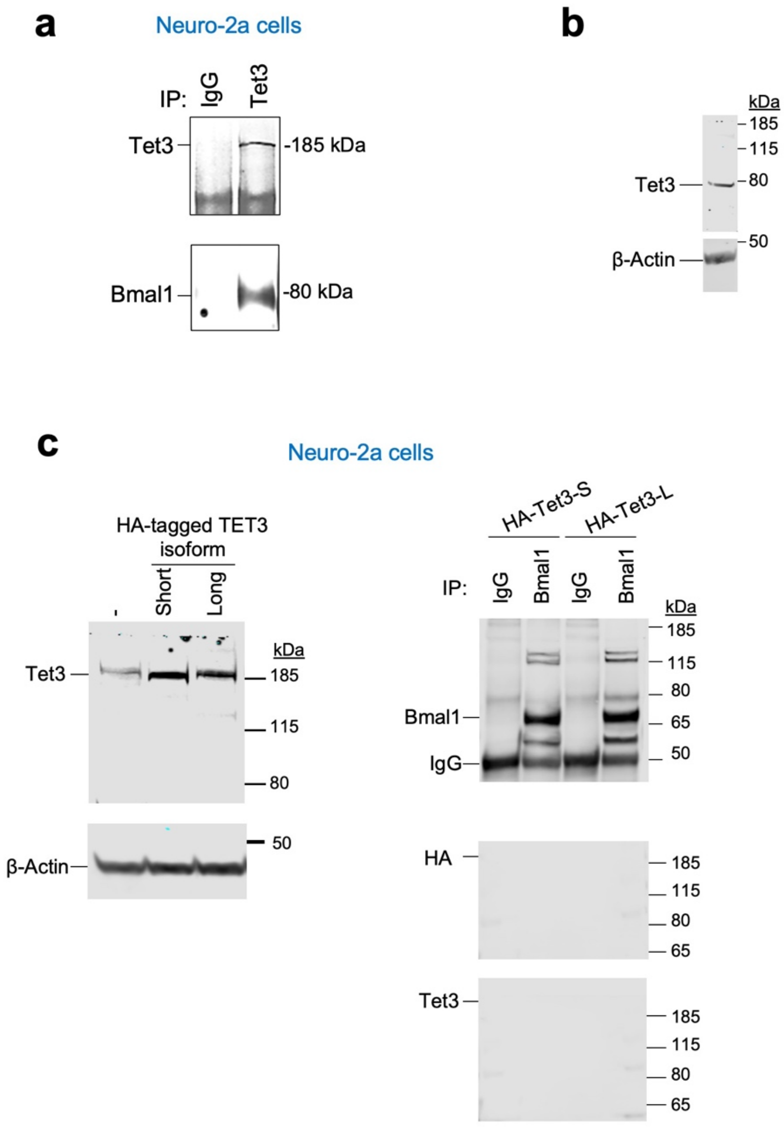

Figure S2. Tet3 interacts with Bmal1.

a. Co-immunoprecipitation (IP) results show interaction of endogenous Bmal1 with Tet3 in Neuro-2a cells. Data representative of three independent experiments.

b. Left, immunoblot of DRG lysate with anti-Tet3 antibody reveals a lower than expected molecular weight fragment of ~80 kDa. Right, Co-immunoprecipitation (co-IP) using anti-Tet3 antibody can pull down Bmal1 in DRG lysate. Note a faint band of Tet3 at 185 kDa, but also multiple bands of smaller weights. Data representative of three independent co-IP experiments.

c. Left, immunoblot of transfected Neuro-2a cells shows overexpression of HA-tagged TET3L or TET3S isoforms. Right, immunoprecipitation (IP) analysis shows that pull-down of Bmal1 fails to co-IP HA-tagged or endogenous TET3 from Neuro-2a lysates.

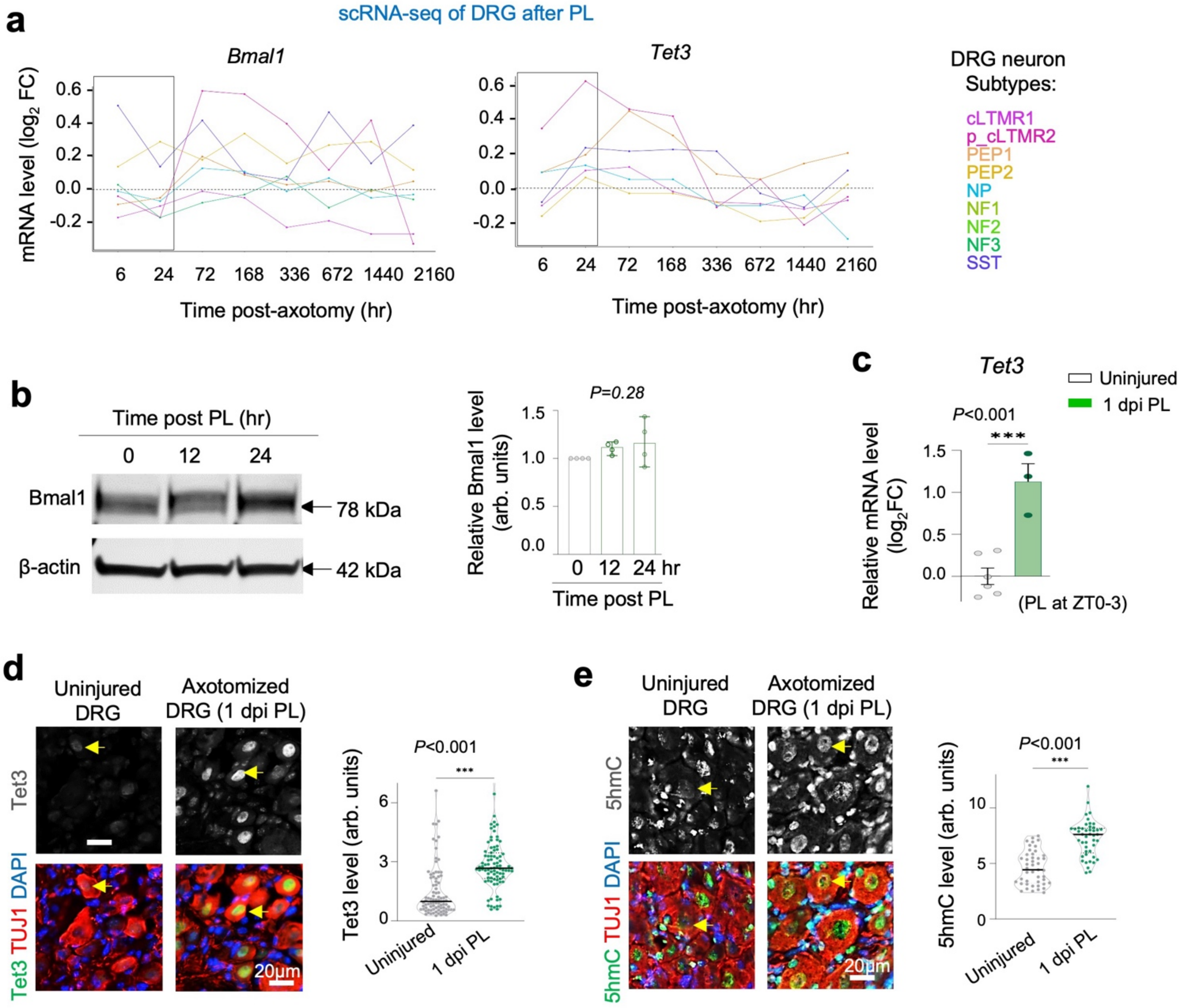

Figure S3. Bmal1 and Tet3 expression are altered in axotomized DRGs.

a. Survey of scRNAseq database of axotomized DRG (Renthal et al. 2019) reveals down-regulation of *Bmal1* (Bmal1) and upregulation of *Tet3* by the majority of neuronal subtypes at 24 hr after PL.

b. Western blot analysis and quantification of Bmal1 protein levels in lumbar DRG tissue lysates at the indicated times post-injury. n=4 DRG samples (L4-6) collected from n=2 mice per group. One-way ANOVA.

c. qRT-PCR shows upregulation of *Tet3* mRNA in lumbar DRGs at 1 d after sciatic nerve crush injury relative to no injury. n=4-6 independent DRG samples per group. Data represent mean ± SEM. Unpaired two-tailed Student's *t*-test.

d, e. IF images and quantifications confirm Tet3 and global 5hmC increase in axotomized DRG neurons at 1 dpi after PL. Violin plots of n=88 (TET3) and n=45 (5hmC) neurons quantified from L4-L6 DRGs of n=3-4 mice per group. Black lines indicate median. Mann-Whitney test.

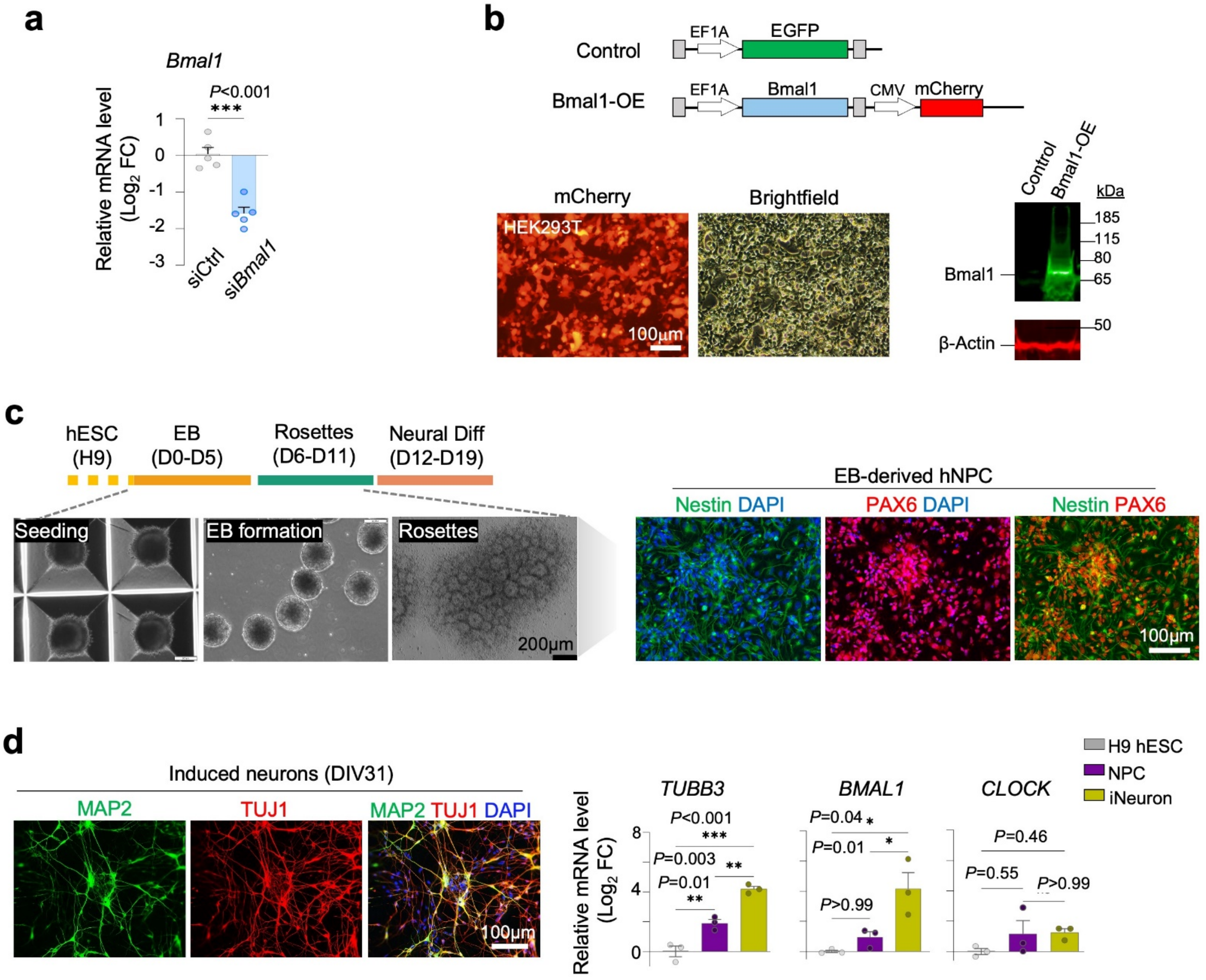

Figure S4. Expression of Bmal1 in induced neurons derived from hES cells.

a. Top, qRT-PCR results demonstrate knockdown of *Bmal1* mRNA by siRNA in Neuro-2a cells. n=5 experiments. Mean ± SEM. Unpaired two-tailed Student's t-test.

b. Left, schematic of lentiviral control vector and vector used to over-express Bmal1. Middle, images of HEK293T cells transfected with Bmal1-OE lentiviral vector. Right, immunoblot analysis of HEK293T lysate confirms Bmal1 overexpression.

c. Top, schematics of generation of induced neurons from hES cells. Middle, bright field images of embryoid body (EB) and Rosette formation. Bottom, IF images confirm expression of markers for NPCs.

d. Representative IF images and qRT-PCR reveal expression of neuronal markers and circadian clock genes in induced neurons, in agreement with a report that circadian clock is operational in NPCs and iNeurons (Mishra et al., 2021). n=3 independent studies. Data represent mean ± SEM. One-way ANOVA with Bonferroni's multiple comparison test.

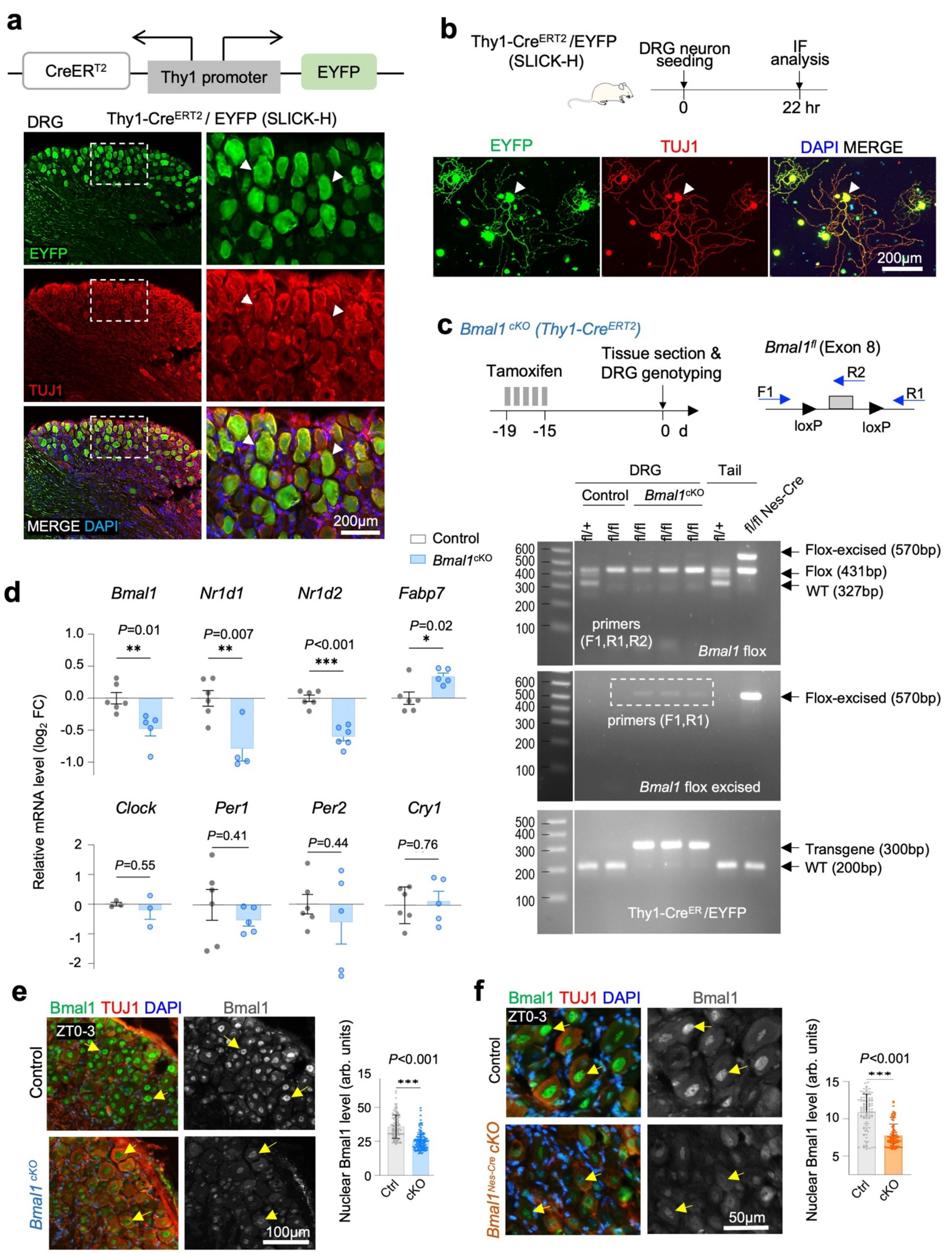

Figure S5. Generation of mutant mice with tamoxifen inducible neuron-specific deletion of *Bmal1*.

a. Top, illustration of bidirectional Thy1 promoter driving the expression of enhanced yellow fluorescent

protein (EYFP) and tamoxifen-inducible Cre-recombinase (CreER^{T2}) in the Thy1-CreER^{T2}/EYFP (SLICK-H) transgenic line. Bottom, representative IF images of lumbar DRGs demonstrate overlap of EYFP and TUJ1 in DRG neurons from Thy1-CreER^{T2}/EYFP mice.

b. Top, experimental paradigm of dissociated DRG neuron culture. Bottom, IF images show overlap of EYFP and TUJ1, validating Thy1 promoter activity in primary DRG neurons.

c. Top left, experimental paradigm to generate *Bmal1* conditional KO (cKO) mice: Tamoxifen injections (on 5 consecutive days; i.p.) were carried out ~2 weeks prior to analysis of DRGs. Top right, diagram of genotyping primers to detect excision of floxed region. Bottom, genotyping results from DRGs, confirming excision of floxed region (primers F1, R1) in *Bmal1* cKO mice. Excision of floxed region in DNA from mouse tail of Nestin-Cre *Bmal1* cKO was used as a positive control. Note that DRG neurons expressing Thy1-CreER^{T2}/EYFP transgene represent ~12% of all cells in DRG tissues (Avraham et al., 2020), while other glial cell types are not affected by Cre activity, explaining the weak PCR signal.

d. qRT-PCR analysis of circadian clock genes in uninjured DRGs neurons. Note expected reduction of *Bmal1* and target genes *Nr1d1* concomitant with increased *Fabp7* in cKO DRGs as previously reported (Musiek et al., 2013). n=3-5 independent studies. Data represent mean ± SEM. Unpaired two-tailed Student's *t*-test.

e, f. Representative IF images and quantification of lumbar DRGs show reduced Bmal1 nuclear protein levels in DRG neurons (TUJ1^{+}) in *Bmal1* cKO mice (both *Bmal1*$^{Thy1\text{-}CreERT2}$ and *Bmal1*$^{Nes\text{-}Cre}$) compared to control. Quantification of top n=110-120 neurons from L5 DRGs isolated from n=3 mice per group at ZT0-3. Data represent mean ± SEM. Mann-Whitney test.

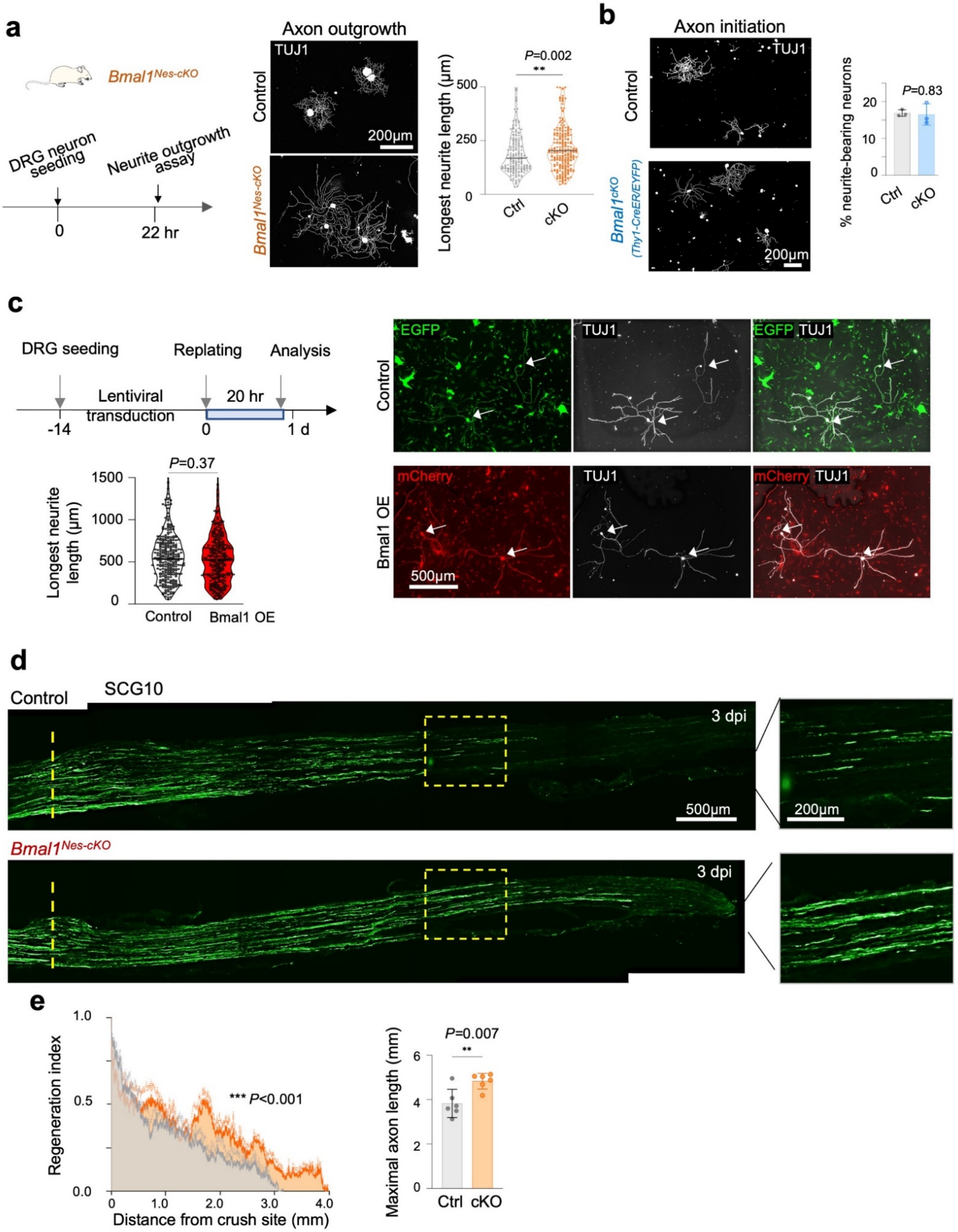

Figure S6. *Bmal1* deletion leads to enhanced neurite outgrowth.

a. Left, experimental paradigm. Right, representative images and quantifications show longer neurite

outgrowth of DRG neurons from *Bmal1*$^{Nes\text{-}cKO}$ mice than controls. Violin plots of n=200-270 neurons from n=3 mice per group. Black lines represent median. Mann-Whitney test.

b. Representative IF images and quantification show comparable proportions of neurite-bearing DRG neurons between genotypes at 22 hr in vitro. n=3 independent cultures per group. Data represent mean ± SEM. Unpaired two-tailed Student's *t*-test.

c. Left experimental paradigm and quantifications of neurite length of DRG neurons overexpressing Bmal1 compared to control. Violin plots of n=280-290 neurons from n=1 mouse per group. Black lines represent median. Mann-Whitney test.

d, e. Representative IF images and quantifications show enhanced axon regeneration (SCG10^{+}) at 3 dpi after sciatic nerve crush injury in *Bmal1*$^{Nes\text{-}cKO}$ as compared to littermate controls. Dashed vertical lines denote lesion center defined by maximal SCG10 immunointensity. Two-way ANOVA for regeneration index. Data represent mean ± SEM. Unpaired two-tailed Student's *t* test for maximal axon length. n=6 mice per genotype.

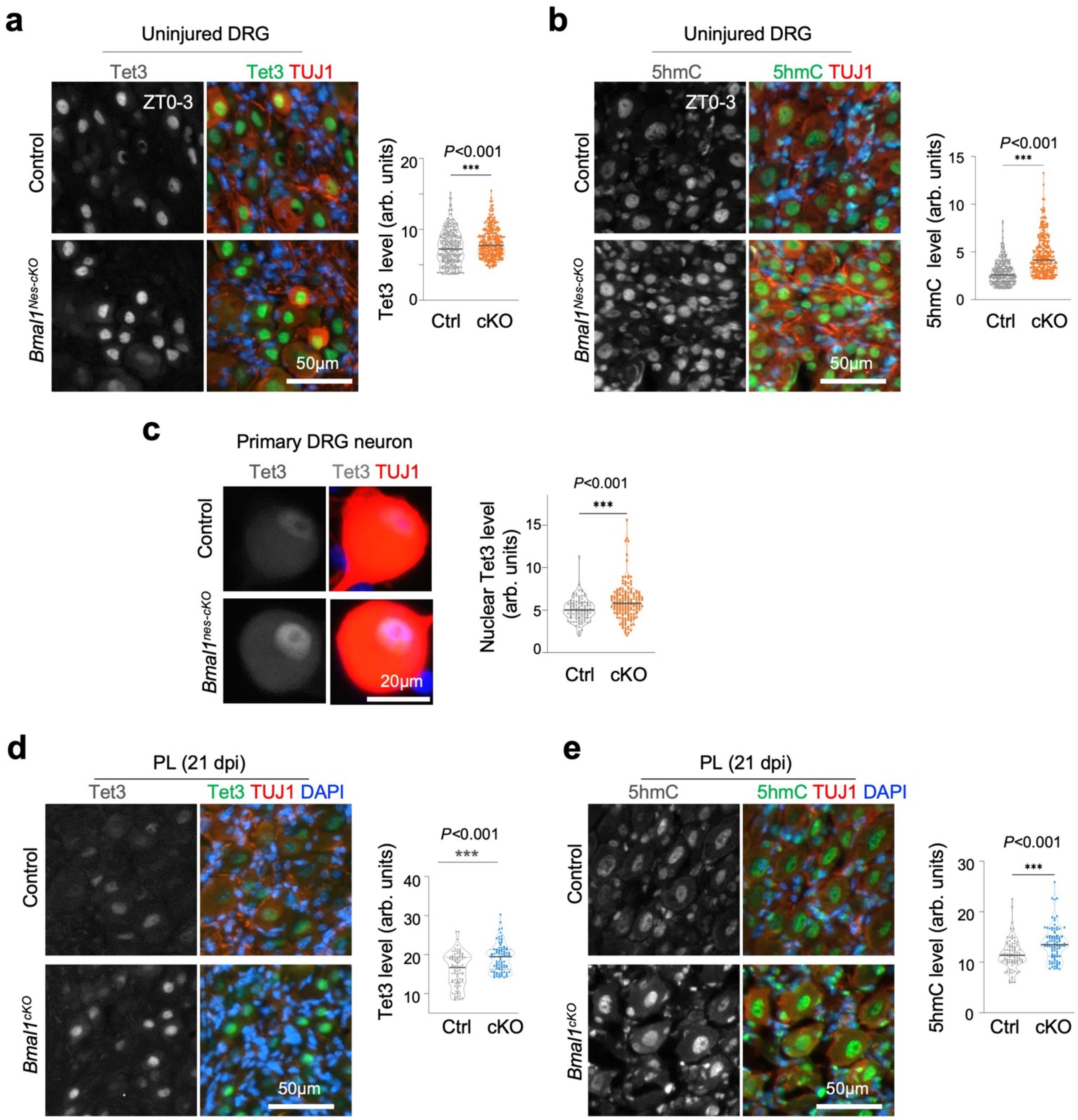

Figure S7. *Bmal1* deletion enhances Tet3 expression and global DNA hydroxymethylation in DRG neurons.

a, b. Representative IF images and quantifications show higher levels of Tet3 and 5hmC in uninjured DRG neurons from $Bmal1^{Nes\text{-}cKO}$ compared to controls. Violin plots of n=300 neurons from L5 DRGs isolated from n=3 mice per group. Black lines represent median. Mann-Whitney test.

c. IF images of primary DRG neurons cultured for 22 hr in vitro show higher Tet3 nuclear immunosignal in neurons from $Bmal1^{Nes\text{-}ckO}$ mice than from controls. Violin plot with mean median value (black line) of n= ~100 neurons isolated from n=3 mice per group. Black lines represent median. Mann-Whitney test.

d, e. Representative IF images and quantifications show sustained elevation of Tet3 (d) and 5hmC (e) levels in axotomized lumbar DRGs at 21 dpi. 90 DRG neurons were quantified from n=9 L4-6 DRGs of n=3 mice per genotype. Black lines represent median. Mann-Whitney test.

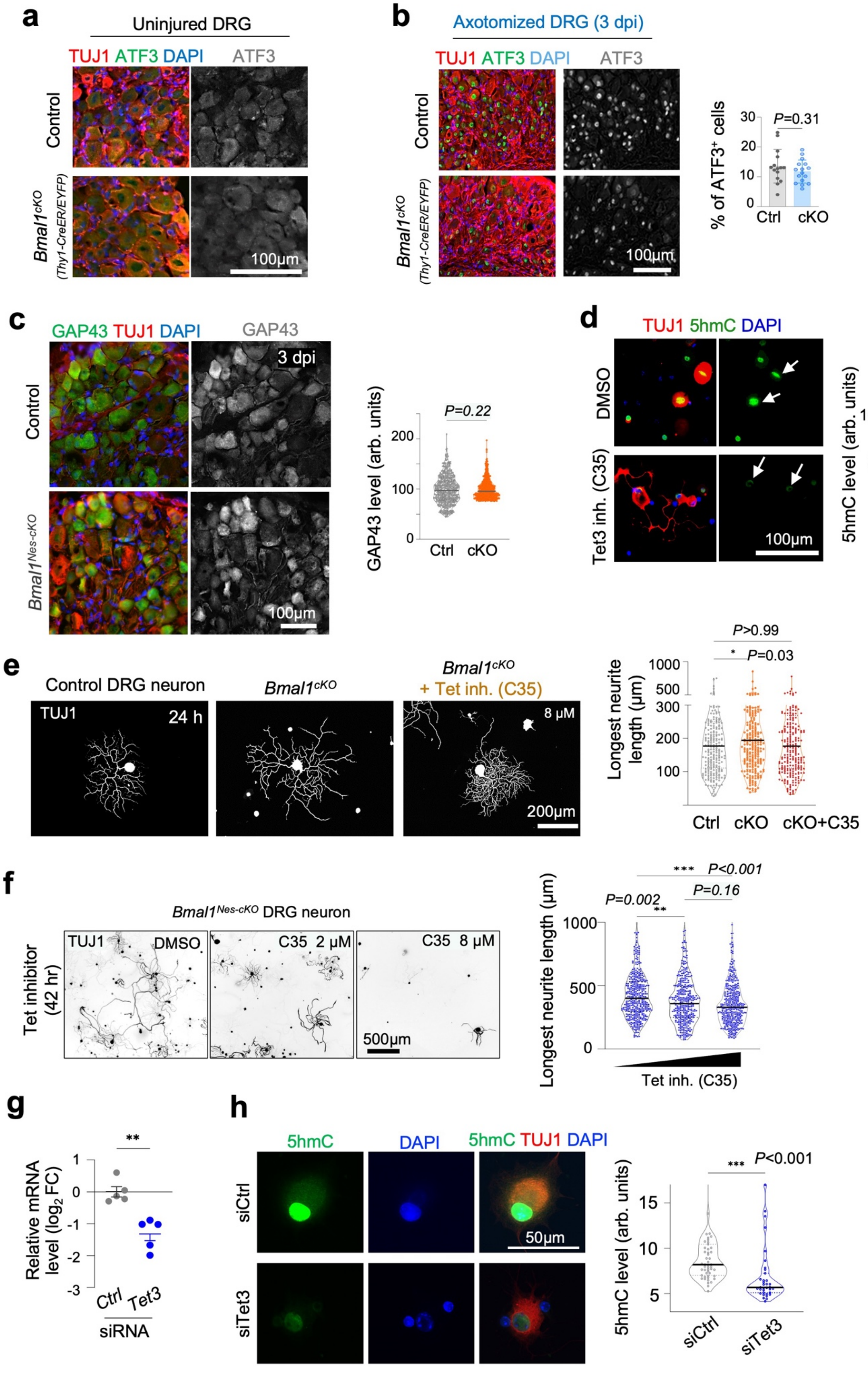

Figure S8. Effect of *Bmal1* deletion on RAGs expression in DRG neurons.

a, b. Representative IF images show low baseline levels of ATF3 expression in uninjured DRGs (a), but robust induction after peripheral injury in either control or *Bmal1*^{cKO} mice (b). Similar IF results were found in n=3 pairs of mice. Quantification of n=16 axotomized DRGs from 6 mice per group. Data represent mean ± SEM. Unpaired two-tailed Student's *t*-test.

c. IF images and quantification of axotomized L4-L6 DRGs at 3 dpi show no change in GAP43 expression in *Bmal1*$^{Nes\text{-}cKO}$ mice compared to control. Violin plots of n= 650 neurons from n=9 DRGs collected from n=3 mice per genotype. Black lines represent median. Mann-Whitney test.

d. IF images and quantification show reduced levels of 5hmC in individual DRG neurons upon treatment with pan-TET inhibitor C35 (8.0 μM). Violin plots of n=170-195 L4-6 DRG neurons for each condition pooled from n=2 mice per group. Black lines represent median. Mann-Whitney test.

e. IF images and quantification of neurite outgrowth at 24 h after plating. Pan-TET inhibitor C35 (8.0 μM) reversed the growth promoting effect of Bmal1 deletion. n= 245-272 neurons for each condition from n=3 mice. Median is shown by black line. Kruskal-Wallis with Dunn's multiple comparison test.

f. IF images and quantification show dose-dependent suppression of neurite length of *Bmal1* cKO DRG neurons upon treatment with pan-TET inhibitor C35. Violin plots of n=373-435 DRG neurons for each condition pooled from n=2 mice. Black lines represent median. Kruskal-Wallis with Dunn's multiple comparison test.

g. qRT-PCR analysis of *Tet3* level in Neuro-2a cells confirms *Tet3* knockdown by siRNA treatment. Data represent mean ± SEM. n=5 independent studies. Unpaired two-tailed Student's *t*-test.

h. IF images and quantification show reduced levels of 5hmC in individual DRG neurons upon siRNA-mediated knockdown of *Tet3*. Violin plots of n=35-51 DRG neurons from L4-6 for each condition. Black lines represent median. Mann-Whitney test.

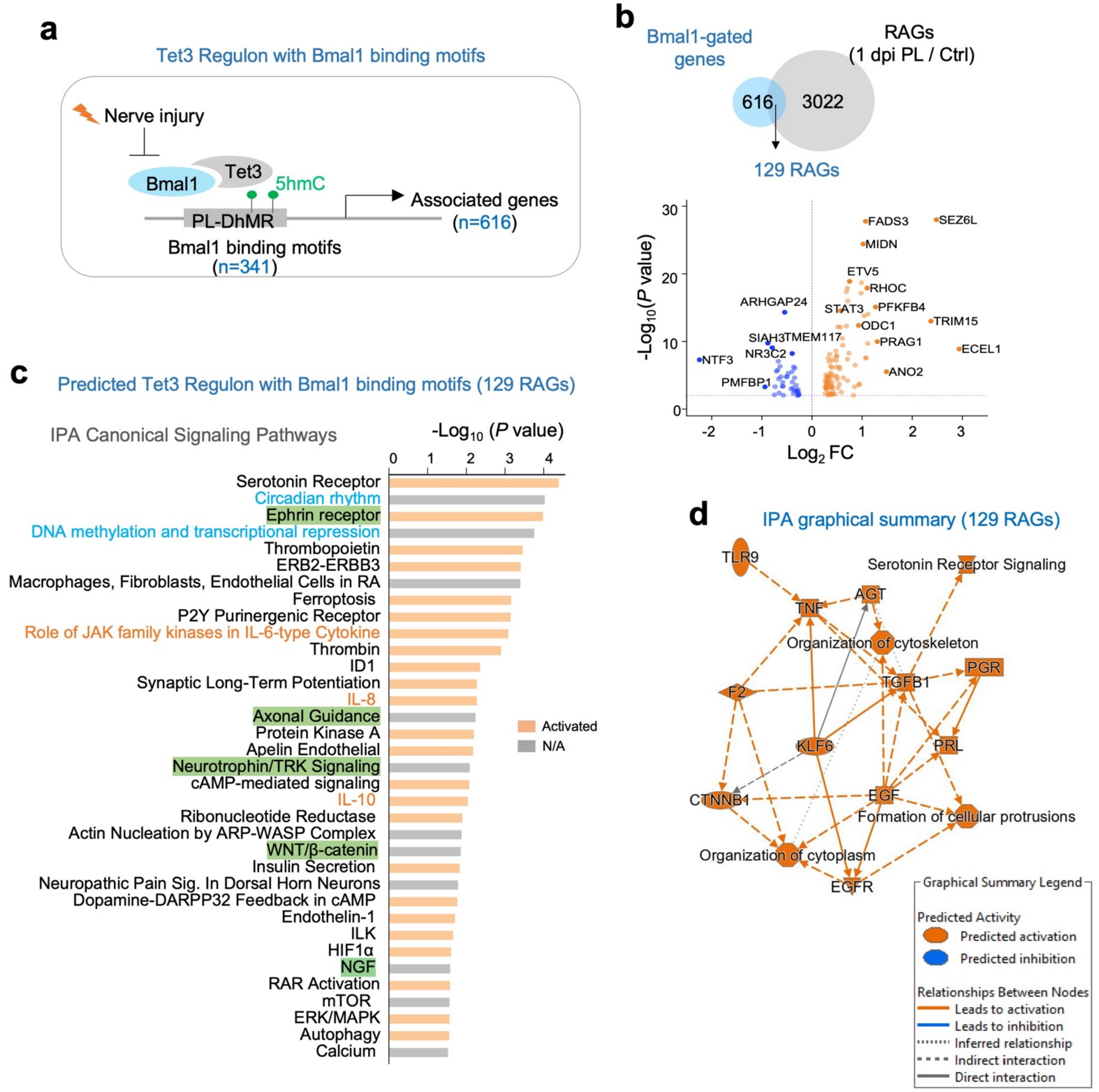

Figure S9. Tet3 regulon containing Bmal1 binding motif in PL-DhMRs is enriched for pathways implicated in axon growth, immune response, and circadian rhythm.

a. Diagram of Tet3-regulon with Bmal1 binding motif within PL-DhMRs and associated genes.

b. Top, Venn diagram shows intersection of RAGs identified by RNA-seq in DRGs at 1 d after PL relative to no injury with genes associated with PL-DhMRs harboring Bmal1 binding motif (n=616). Bottom, volcano plot of the 129 overlapping RAGs, with labeled top DEGs.

c. IPA analysis for the top enriched canonical pathways of the 129 RAGs harboring Bmal1 binding motif in PL-DhMRs. Circadian rhythm, DNA methylation, neurotrophin and cAMP signaling, axon guidance, immune response, Wnt/β-catenin, and HIF1A signaling pathways are involved. Orange bars: enriched pathways with activation; grey bars: insufficient evidence for prediction of activation state. Right-tailed Fisher's exact test.

d. IPA graphical summary of top enriched signaling pathways and major biological themes of the 129 overlapping RAGs.

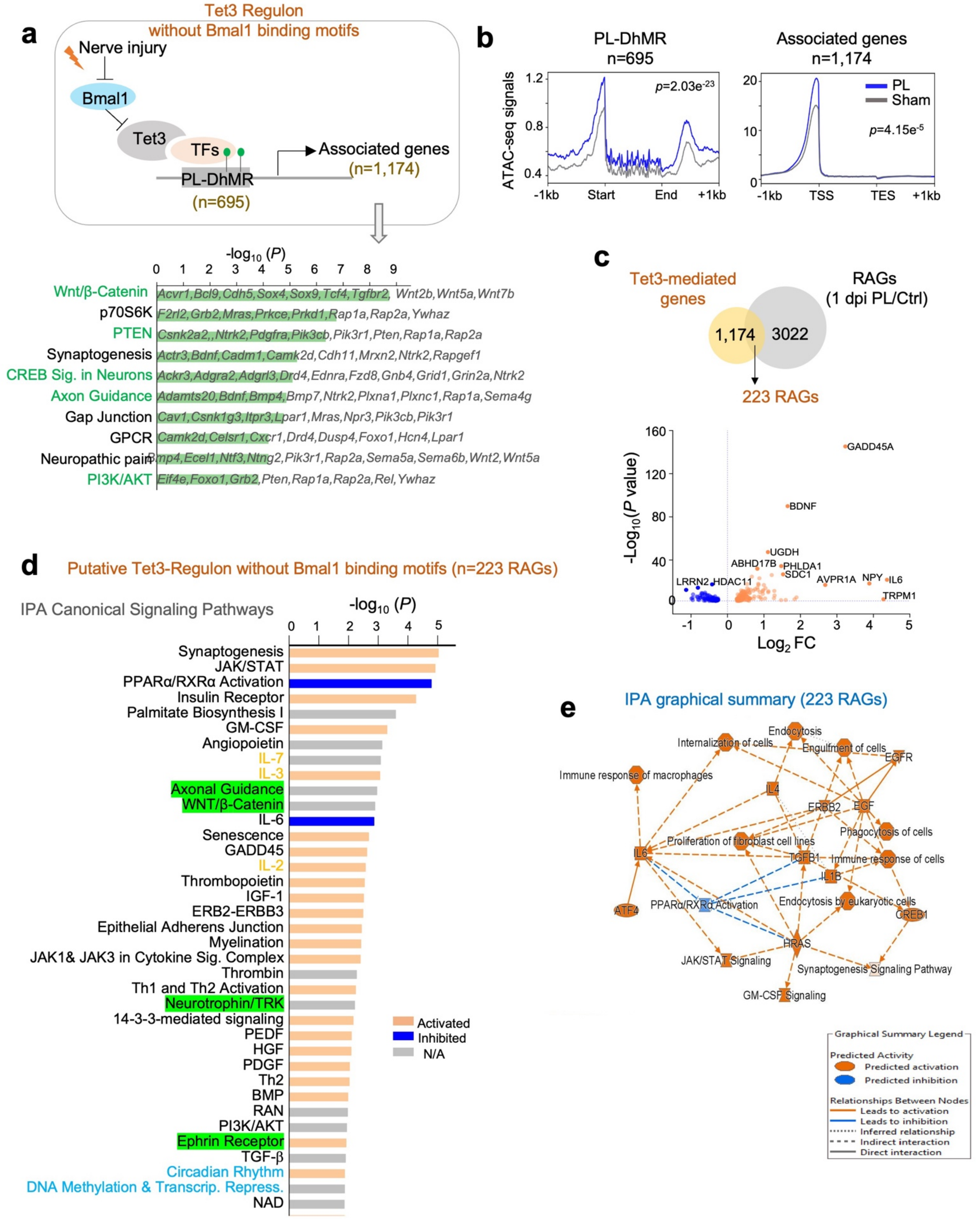

Figure S10. Tet3 regulon without Bmal1 binding motif in PL-DhMRs concerns axon growth and immune response to axonal injury.

a. Top, diagram of Tet3-regulon without Bmal1 binding motif within PL-DhMRs (n=695) and associated genes

(n=1,174). Bottom, top enriched signaling pathways in the 1,174 genes harboring PL-DhMRs without Bmal1 binding motif by IPA. Right-tailed Fisher's exact test.

b. Analysis of ATAC-seq signals from PL-DhMRs (left) and at the TSS of the associated genes (right) shows increased chromatin accessibility after PL at 1 dpi compared to sham surgery. TSS, transcription start site; TES, transcription end site. Original ATAC-seq data from Palmisano et al., 2019. Welch's two-sample t-test.

c. Top, Venn diagram shows intersection of RAGs identified by RNA-seq in DRGs at 1 d after PL relative to no injury with the PL-DhMRs associated genes without Bmal1 binding motif (n=1,174). Bottom, volcano plot of 223 overlapping RAGs, with labeled top DEGs.

d. IPA for the top enriched canonical pathways of the 223 overlapping RAGs associated with PL-DhMRs demonstrates enrichment of immune response and growth factor signaling. Orange and blue bars depict pathway activation and inhibition, respectively, and grey bars indicate insufficient evidence for prediction of activation state. Right-tailed Fisher's exact test.

e. IPA graphical summary analysis of the 223 overlapping RAGs associated with PL-DhMRs highlights immune response, phagocytosis/endocytosis, and CREB1 signaling as major biological themes.

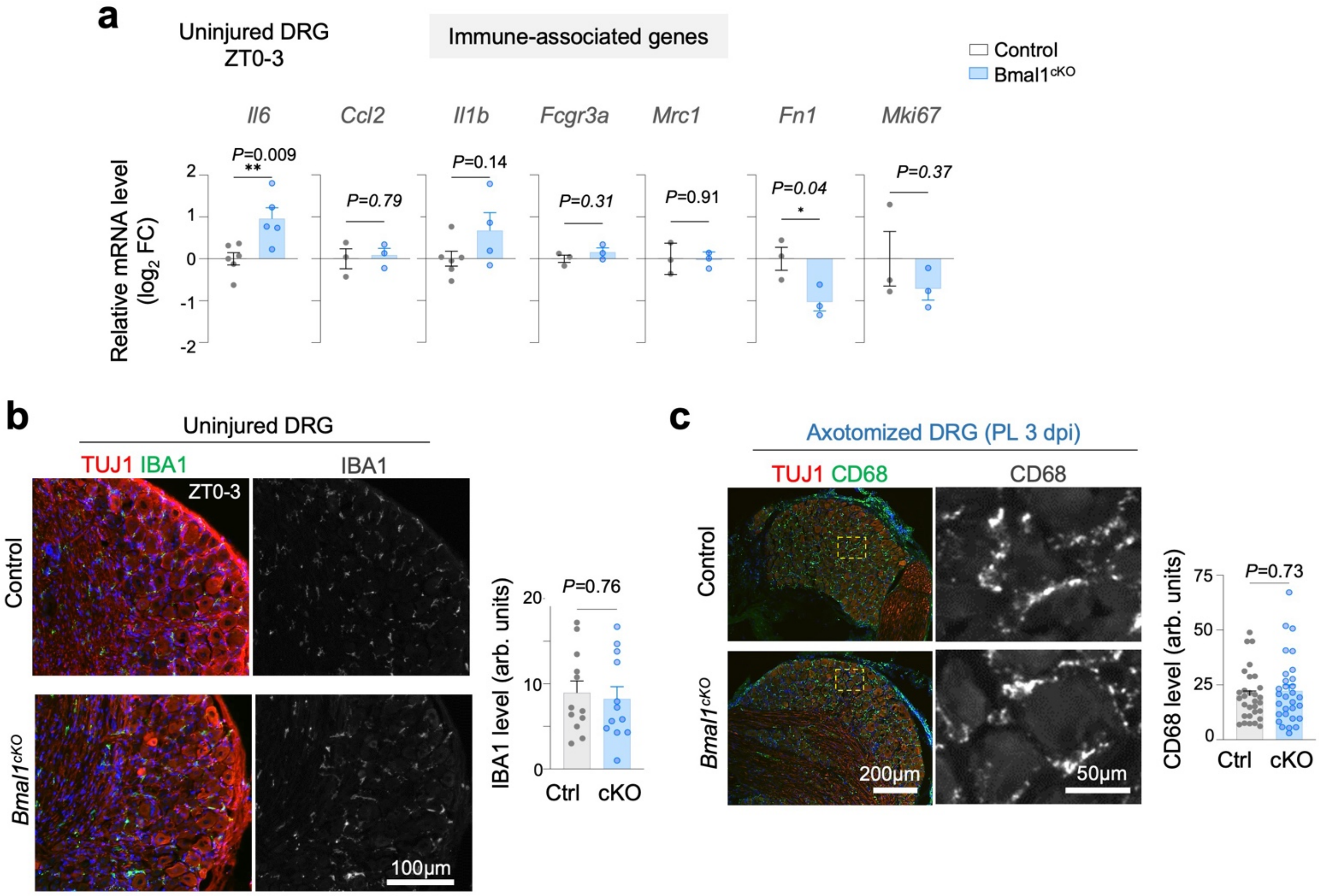

Figure S11. Neuron-specific *Bmal1* cKO does not significantly affect immune genes in uninjured DRGs.

a. qRT-PCR results show comparable mRNA levels of immune related genes in uninjured DRGs from *Bmal1* cKO mice vs. littermate controls collected at ZT0-3. Data represent mean ± SEM. n=3-5 mice per genotype, with 6-9 DRGs pooled per mouse. Unpaired two-tailed Student's *t*-test.

b. IF images and quantifications show no significant changes of Iba1 expression in uninjured DRGs between genotypes. Bars represent mean ± SEM. Quantifications from 2 randomly selected areas per DRG, n=6 DRGs collected from 3 mice per group at ZT0-3. Unpaired two-tailed Student's *t*-test.

c. IF images and quantification show no significant changes of CD68 expression in axotomized DRGs at 3 d after PL. Quantification from 2 randomly selected areas per DRG, n=15-16 DRGs pooled from 6 mice per group with 3 collected at ZT0-3 and remaining at ZT12-15. Bars represent mean ± SEM. Unpaired two-tailed Student's *t*-test.

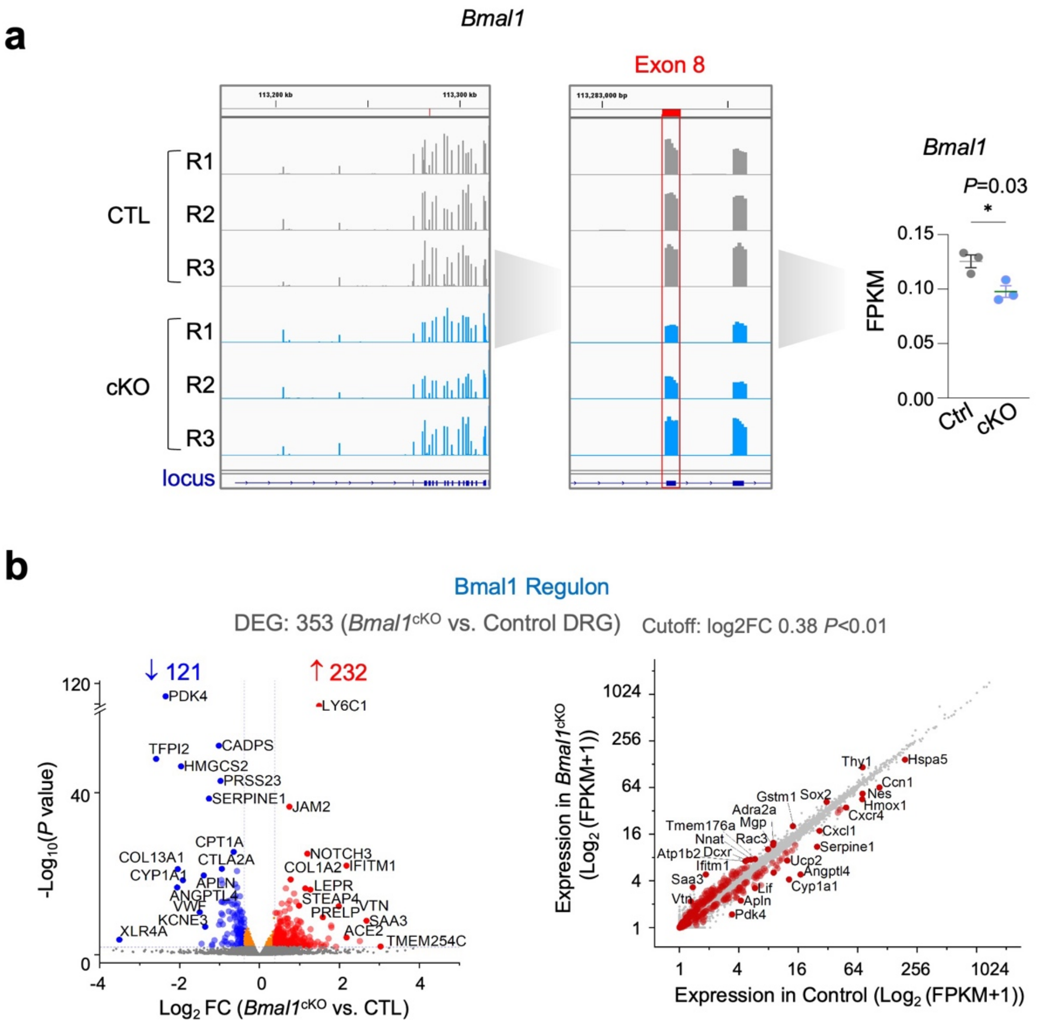

Figure S12. Bmal1-dependent genes in regenerating DRG neurons after peripheral axotomy.

a. Left**,** RNA-seq coverage tracks for *Bmal1* from primary DRG neurons at 28 hours-post seeding. Floxed exon 8 is highlighted (red box) showing reduced coverage in *Bmal1* cKO compared to control. Right, absolute expression level of *Bmal1* as FPKM. Data represent mean ± SEM. n=3 samples each. Unpaired two-tailed Student's t-test.

b. Left, volcano plot of DEGs in *Bmal1* cKO DRG neurons vs. control reveals nearly twice as many upregulated than downregulated genes, cutoffs: 0.38 log_2FC and $P < 0.01$. Top DEGs are labeled. Right, scatter plot of genes by absolute expression levels in control (x-axis) versus *Bmal1* cKO DRG neurons (y-axis) at 1-day post-axotomy. DEGs are indicated in red.

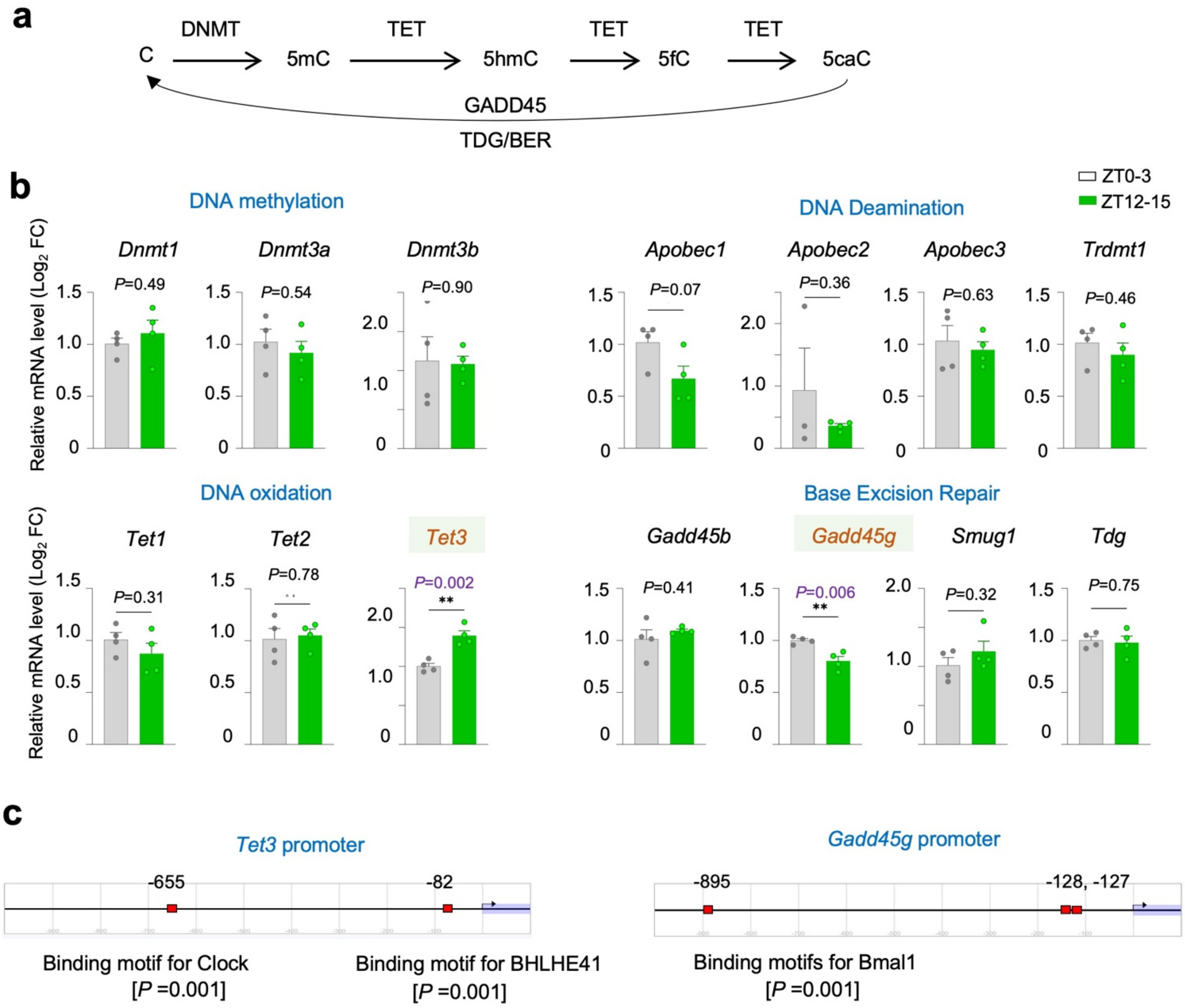

Figure S13. Expression of genes involved in DNA (de) methylation pathways at different ZTs.

a. Schematic of DNA methylation/demethylation pathway. Cytosine (C) methylation is mediated by DNA methyltransferase (DNMT). Methylated cytosine (5mC) is converted to 5-hydroxymethylcytosine (5hmC), 5-formylcytosine (5fC), and 5-carboxylcytosine (5caC) by iterative oxidation catalyzed by TETs. Complete demethylation is mediated by the thymine-DNA glycosylase (TDG) / Base Excision Repair pathway (BER), which recognizes either 5fC or 5caC as substrates. GADD45 interacts with TDG to promote cytosine demethylation.

b. qRT-PCR results of DNA (de)methylation pathway genes in uninjured DRGs isolated at ZT0-3 or ZT12-15 show that only *Tet3* and *Gadd45g* exhibit diurnal transcriptional changes in DRGs. Data represent mean ± SEM. n= 7-9 DRGs isolated from 3-4 mice per group. Unpaired two-tailed Student's *t*-test.

c. Analysis for transcription factor binding sites in *Tet3* and *Gadd45g* promoter regions, performed with Eukaryotic Promoter Database (EPD) platform of Expasy (SIB Swiss Institute of Bioinformatics).

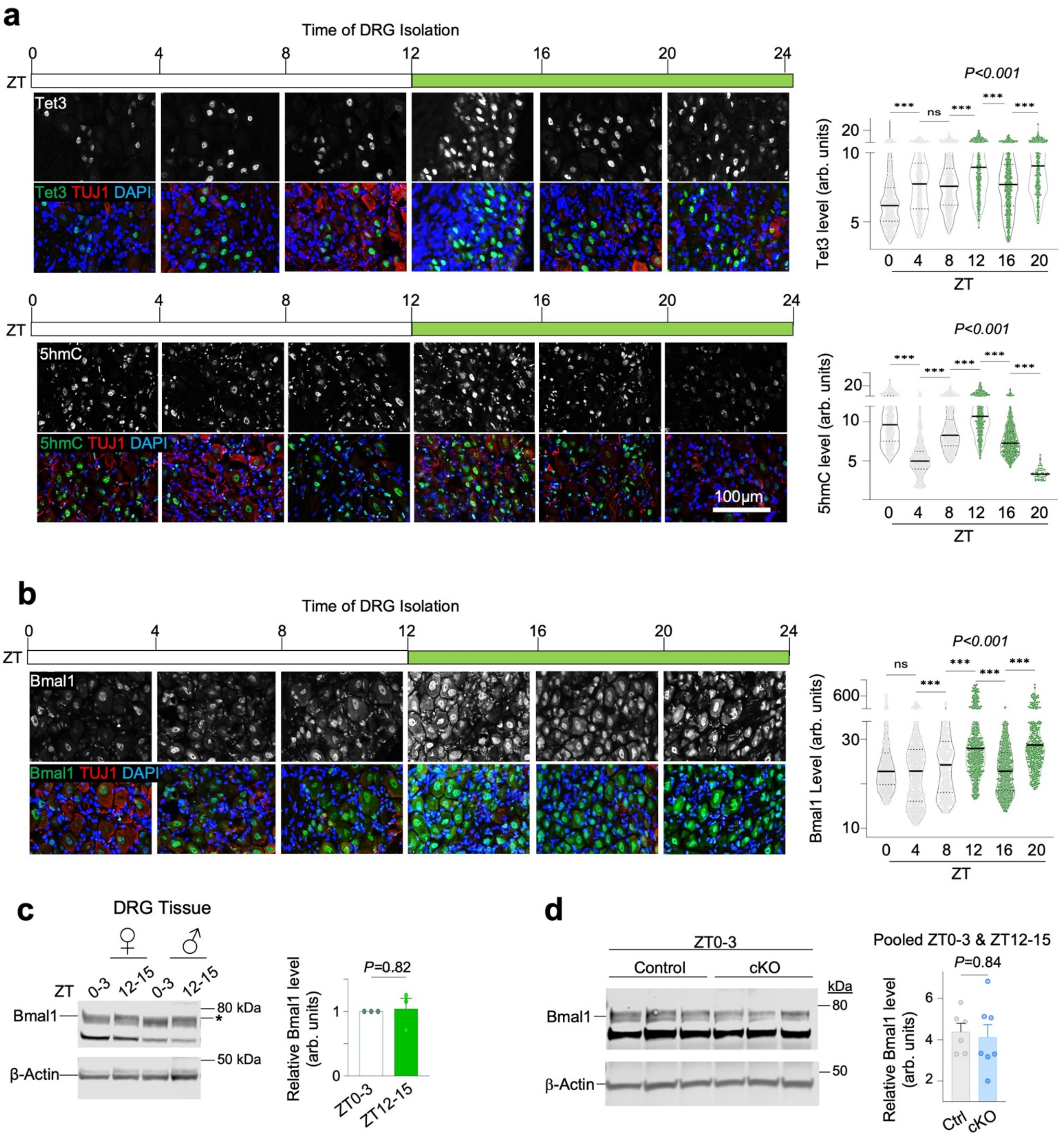

Figure S14. Analysis of peak time-of-day expression of Bmal1 and Tet3-5hmC in DRGs.

a, b. Representative IF images and quantifications of Tet3 and 5hmC levels (a) and Bmal1 (b) in uninjured DRG neurons at 4-hour intervals over 24 hrs. Note lag time of Bmal1 protein level relative to mRNA as previously reported (Lee et al., 2001; Tamaru et al., 2003). Median is shown by black line. n=9 DRGs isolated from 3 mice per condition. Two-way ANOVA followed by Bonferroni's multiple comparison test. *P* values **P*<0.05, ***P*<0.01, ****P*<0.001, and ns, nonsignificant.

c. Immunoblot analysis and quantification of Bmal1 protein levels in lumbar DRG tissue lysates (from male or female) show comparable levels at different ZT. Note post-translational modification of Bmal1 reproducibly observed at ZT12-15 (asterisk). n=3 independent experiments. Data represent mean ± SEM. Unpaired two-

tailed Student's *t*-test.

d. Immunoblotting and quantification show no significant reduction of Bmal1 protein level in DRG tissues from cKO mice relative to controls. Pooled quantification of n=6-7 mice per group. Data represent mean ± SEM. Mann-Whitney test.

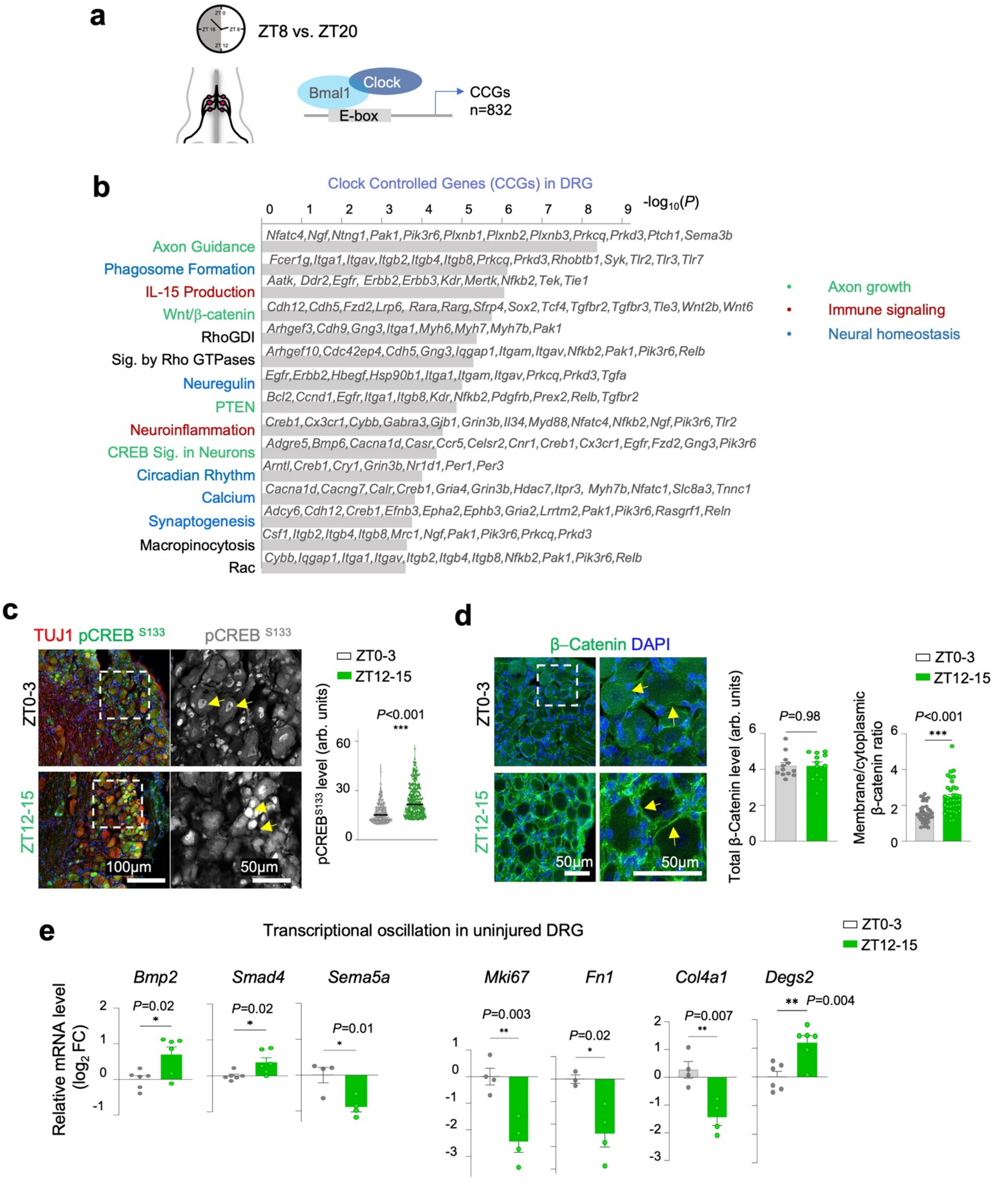

Figure S15. DRG clock-controlled genes are implicated in pathways concerning axon growth, immune signaling, and neural homeostasis.

a. Schematic diagram: RNA-seq study of lumbar DRGs at ZT8 vs. ZT20 identified 832 clock-controlled genes (CCGs) (Kim et al., 2020).

b. IPA of DRG CCGs (n=832) show the top 15 enriched pathways (individual CCGs indicated on the right).

Right-tailed Fisher's exact test.

c. Validation study by IF images and quantification verified higher nuclear pCreb^{S133} levels in uninjured DRG neurons (TUJ1^{+}) at ZT12-15 than ZT0-3. Violin plots of top 300 neurons from 6 lumbar DRGs dissected from n=4 pairs of mice. Median is shown by black line. Mann-Whitney test.

d. Representative IF images and quantification show higher membrane β-catenin levels in uninjured DRG neurons (TUJ1^{+}) at ZT12-15 than ZT0-3. Total β-catenin data from n=2-3 randomly selected areas of lumbar DRGs from n=4 mice per group. Ratio of membrane to cytoplasmic β-catenin data from 9 random areas of lumbar DRGs from n=4 pairs of mice. Data represent mean ± SEM. Unpaired two-tailed Student's *t*-test.

e. qRT-PCR survey of transcription levels of selected CCGs in DRGs at ZT0-3 and ZT12-15 confirmed diurnal changes. 6-9 pooled DRGs per mouse, n=3-6 mice per group. Data represent mean ± SEM. Unpaired two-tailed Student's *t*-test.

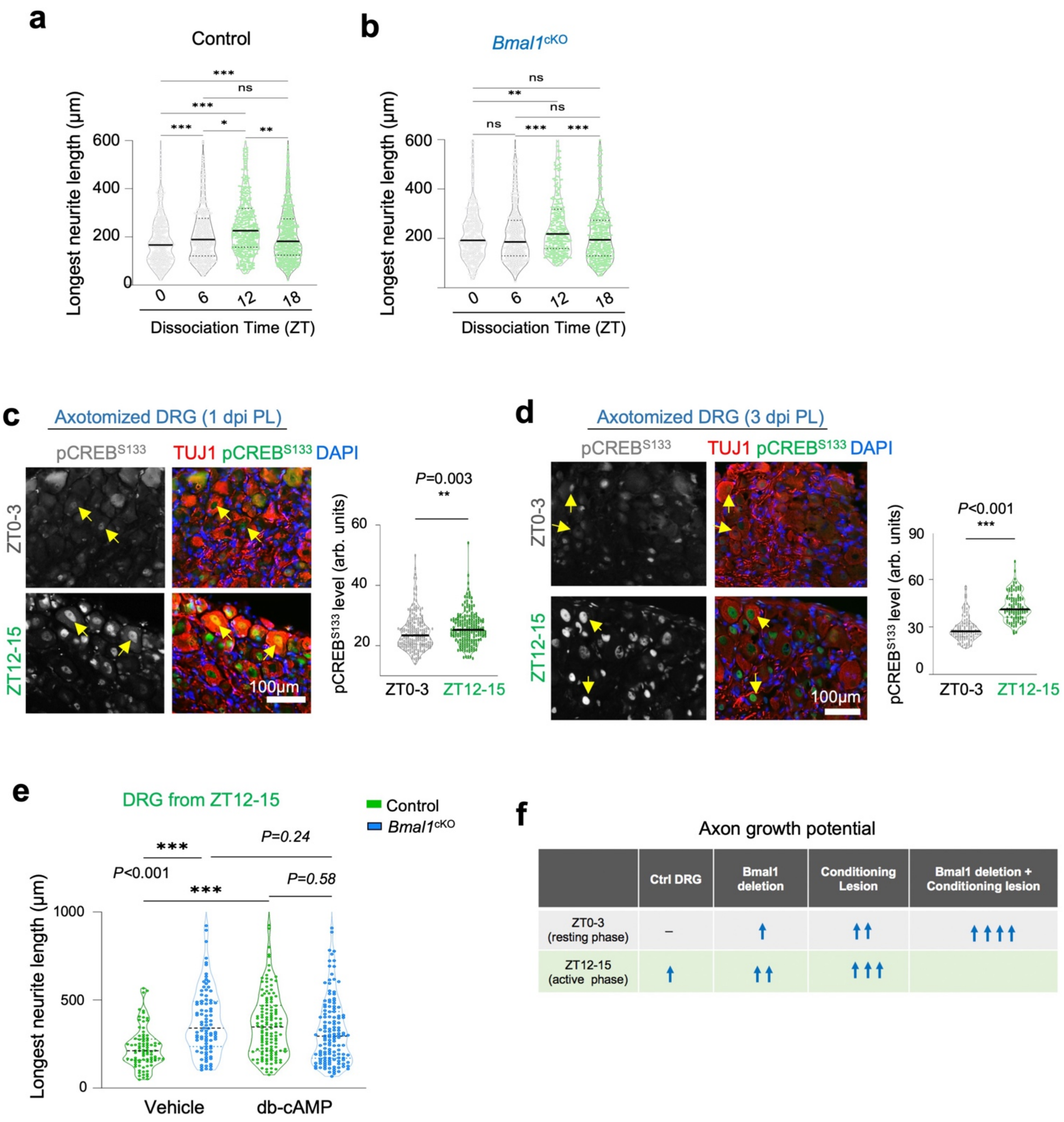

Figure S16. Time of day effect of Bmal1 deletion on neurite outgrowth and cAMP-CREB pathway activation after PL.

a, b. Quantifications on time-of day effect on neurite outgrowth capacity shows sustained diurnal rhythmicity of control (a) and *Bmal1* cKO neurons (b). For each ZT, at least n=300 DRG neurons were quantified from n=2-3 mice per group. Median is shown by black line. Two-way ANOVA followed by Bonferroni's multiple comparison test. *P* values **P*<0.05, ***P*<0.01, ****P*<0.001, and ns, nonsignificant.

c, d. IF images and quantifications show higher nuclear pCreb^{S133} levels in axotomized DRG neurons (TUJ1^{+}) at 1 or 3 dpi when injury had occurred at ZT12-15 than at ZT0-3. Violin plots of n=190 neurons (1 dpi) or 100 neurons (3 dpi) from L5 lumbar DRG dissected from n=4 pairs of mice. Median is shown by black line. Mann-Whitney test.

e. Quantification of neurite outgrowth from DRG neurons isolated at ZT12-15 from *Bmal1* cKO or control

mice and treated with vehicle or 1 mM db-cAMP (vehicle data also shown in Fig. 10D). Note that db-cAMP treatment did not further augment the enhanced neurite outgrowth capacity of *Bmal1* cKO neurons. n=88-136 neurons per genotype. Median is shown by black line. Two-way ANOVA followed by Bonferroni's multiple comparison test.

f. Tabular summary of the effects of Bmal1 deletion and conditioning lesion on axon regeneration. Bmal1 deletion mimics but does not match the conditioning lesion, which likely operates through other pro-regenerative mechanisms. Remarkably, Bmal1 deletion can further augment the conditioning effect, which only causes a transient downregulation of Bmal1.

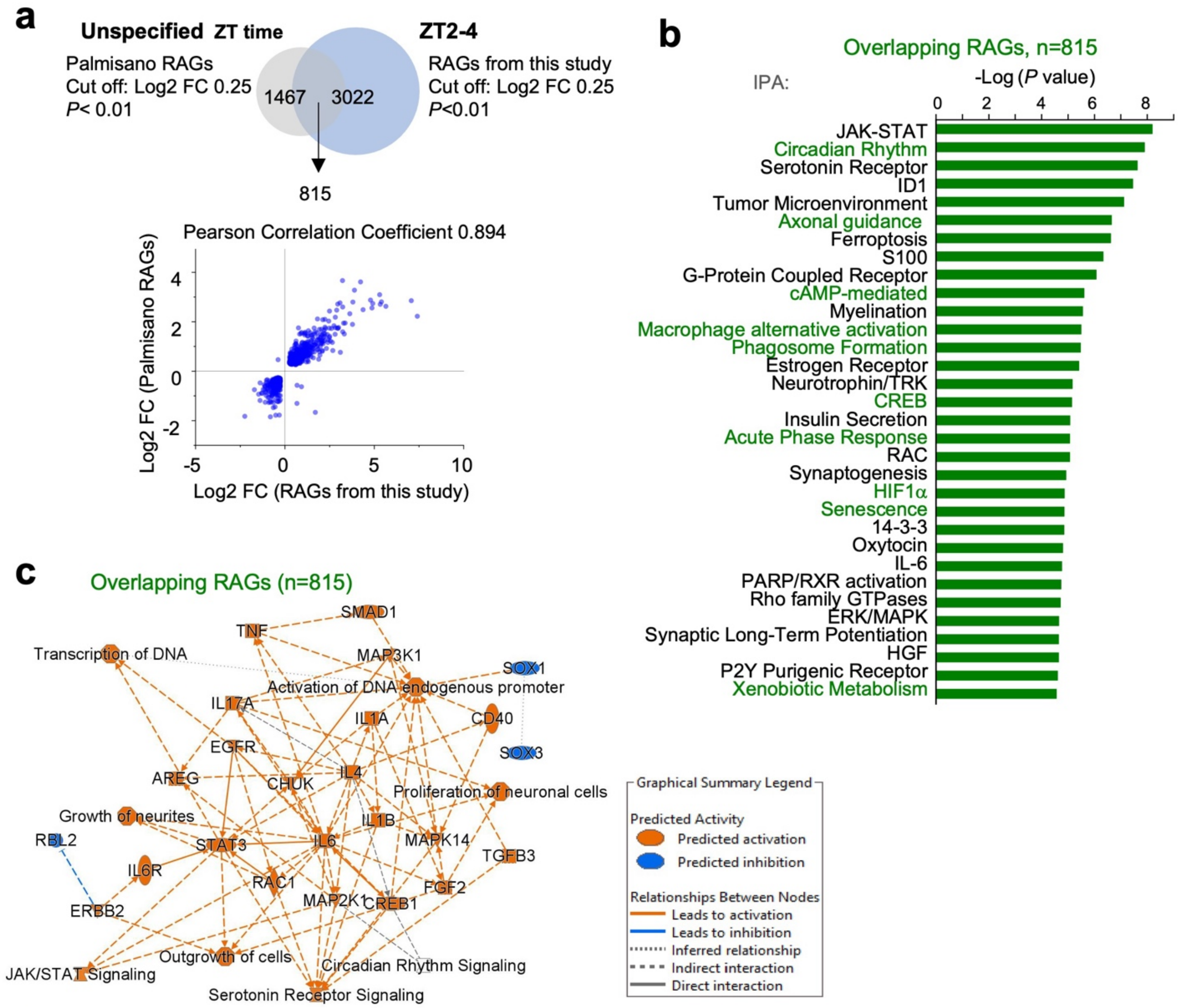

Figure S17. Time-of-day effect on regeneration-associated genes in axotomized DRG after PL

a. Top, Venn diagram shows intersection of RAGs identified by our study (n=3,022) versus Palmisano et al. dataset (n=1,467). Bottom, volcano plot of 815 overlapping RAGs, with labeled top differentially regulated genes.

b. IPA for canonical pathways of the 815 overlapping RAGs demonstrate top enrichment of pathway activated in *Bmal1* cKO mice (refer to main Fig. 7d, f, g). Highlighted pathways in green are shared with *Bmal1* cKO canonical pathway enrichment results. Right-tailed Fisher's exact test.

c. IPA graphical summary of the 815 overlapping RAGs highlights circadian rhythm, neurite growth, transcriptional activation, and immune response as top relevant pathways.

Uncropped Western blots of Supplementary Figures

Figure S2a

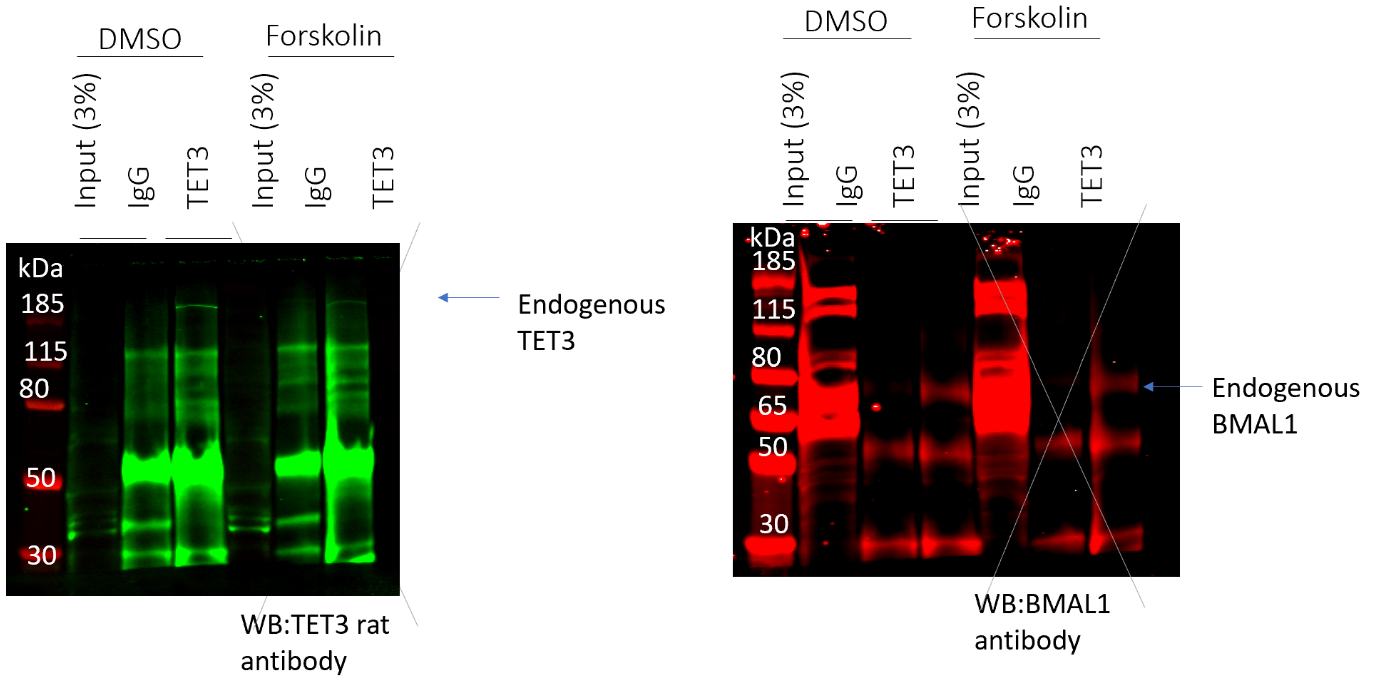

Figure S2b

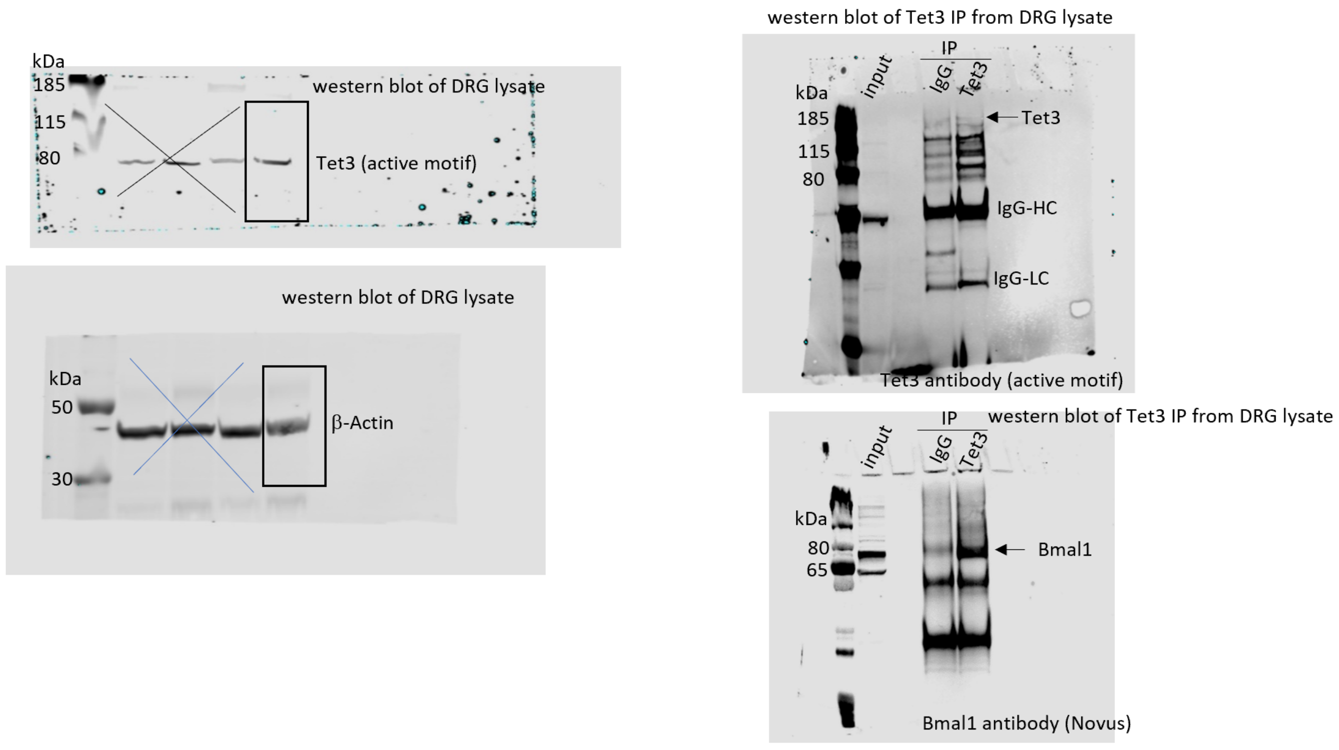

Figure S2c

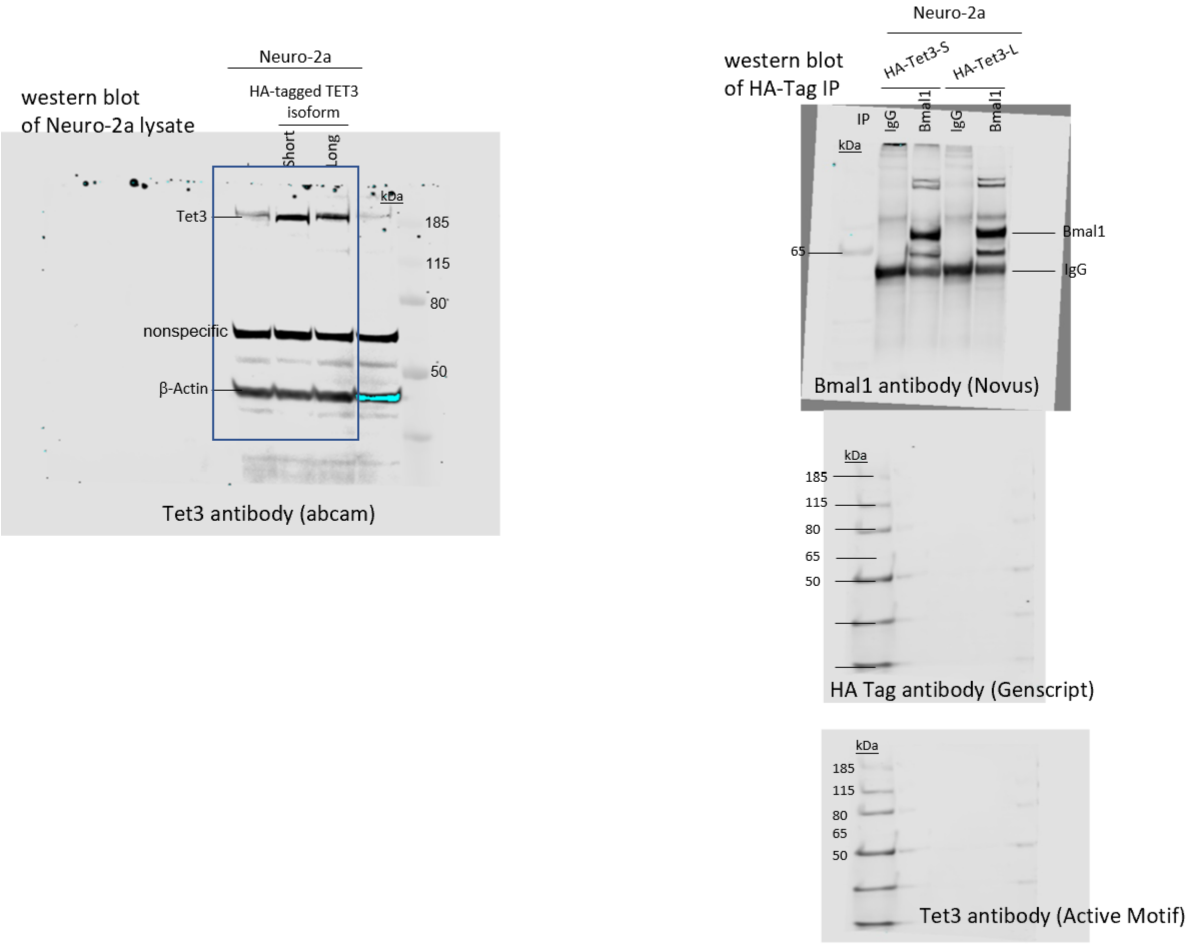
western blot
of Neuro-2a lysate
Neuro-2a
HA-tagged TET3
isoform
Short
Long
kDa
Tet3
185
115
80
nonspecific
50
β-Actin
Tet3 antibody (abcam)
western blot
of HA-Tag IP
Neuro-2a
HA-Tet3-S
HA-Tet3-L
IP
IgG
Bmal1
IgG
Bmal1
kDa
65
Bmal1
IgG
Bmal1 antibody (Novus)
kDa
185
115
80
65
50
HA Tag antibody (Genscript)
kDa
185
115
80
65
50
Tet3 antibody (Active Motif)

Figure S3b

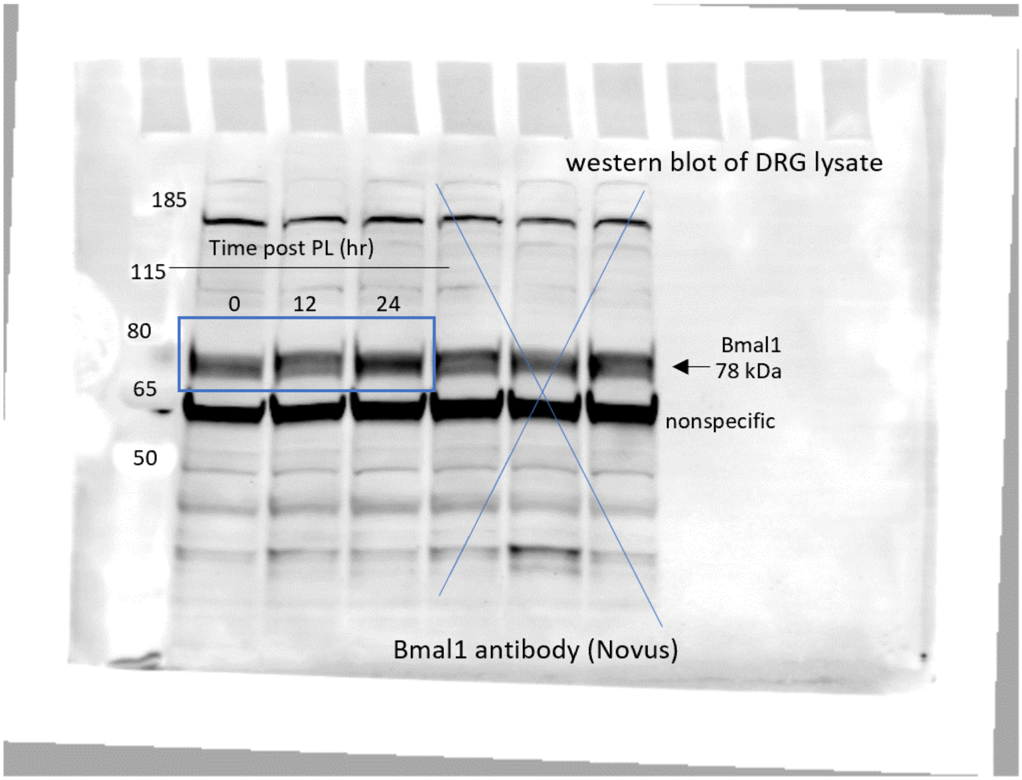

Figure S4d

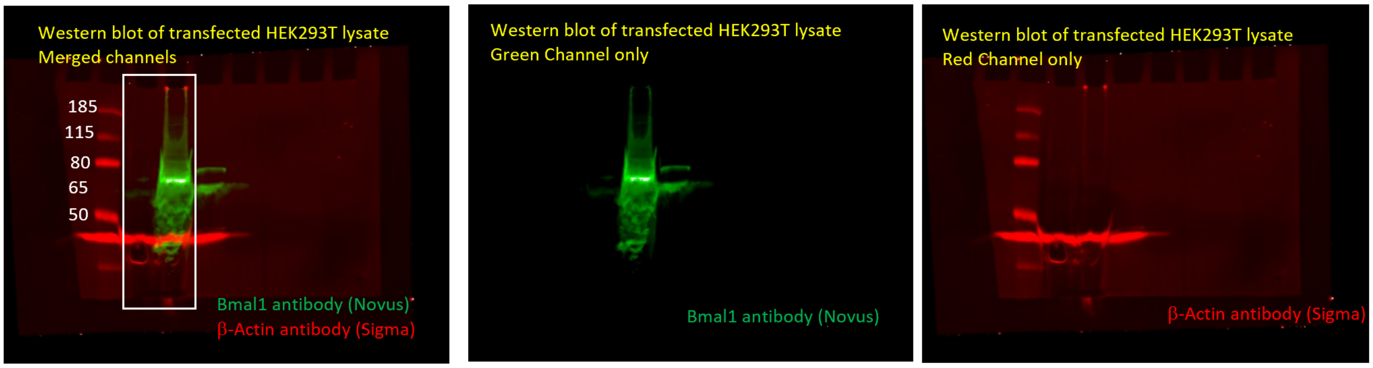

Figure S14c

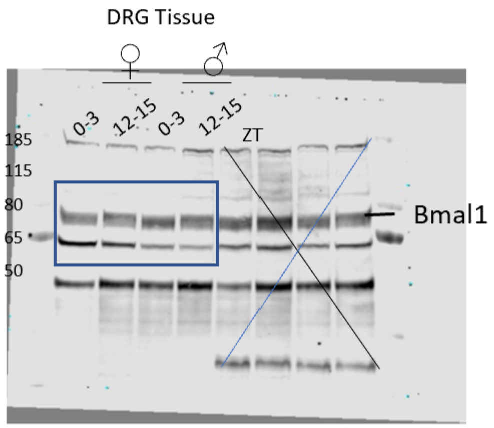

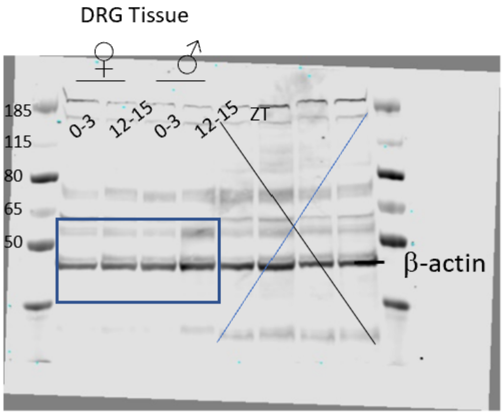

Figure S14d

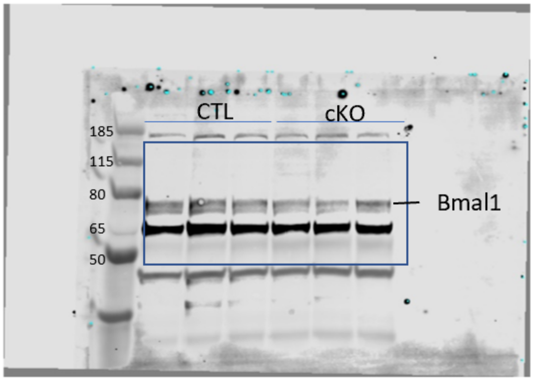

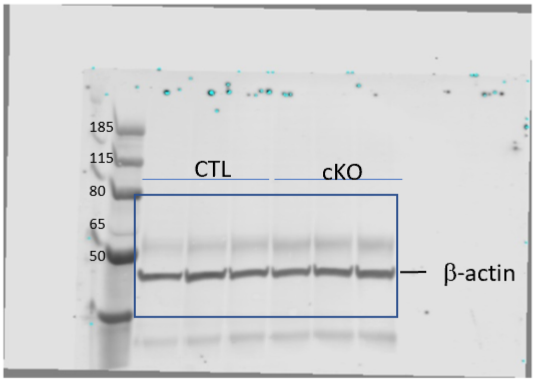

Supplement: Supplementary file 2 — Supplementary Information [file 41467_2023_40816_MOESM2_ESM.pdf]
