# Supplementary material for: Circadian clock regulator Bmal1 gates axon regeneration via Tet3 epigenetics in mouse sensory neurons
Source: Nat Commun. 2023 Aug 24;14:5165. doi: 10.1038/s41467-023-40816-7 (PMC10449865; doi:10.1038/s41467-023-40816-7)
Supplement: Supplementary file 3 — Description of Additional Supplementary Files Document [file 41467_2023_40816_MOESM3_ESM.pdf]

Description of Additional Supplementary Files

Supplementary Data 1. Transcription factor binding motif enrichment in PL-DhMRs.

Supplementary Data 2. Genomic coordinates of PL-DhMRs with 5hmC gain or loss and associated genes.

Supplementary Data 3. Genomic coordinates of PL-DhMRs with Bmal1 binding motif and associated genes.

Supplementary Data 4. RAGs (PL vs. no injury, ZT2-4) at 1 dpi (cutoff 0.25 log2FC, $P<0.01$, n=3,022) based on RNA-seq. P value determined by Welch's two-sample t-test.

Supplementary Data 5. Intersection of RAGs with genes associated with PL-DhMRs (n=346).

Supplementary Data 6. RAGs with Bmal1 binding motif within PL-DhMRs (n=129).

Supplementary Data 7. RAGs without Bmal1-motif within in PL-DhMRs (n=223).

Supplementary Data 8. Genomic coordinates of PL-DhMRs without Bmal1 binding motif and associated genes.

Supplementary Data 9. Bmal1 regulon in DRG neurons (Bmal1cKO vs. control) at 28 hr postseeding (cutoff of 0.25 log2 FC, $P<0.01$, n=625 DEGs). P value determined by Welch's twosample t-test.

Supplementary Data 10. Bmal1-regulated genes with PL-DhMRs (n=85 genes). P value determined by Welch's two-sample t-test.

Supplementary Data 11. Bmal1-regulated genes with Bmal1 binding motif within PL-DhMRs (n=36 genes). P-value determined by Welch's two-sample t-test.

Supplementary Data 12. Clock controlled genes in DRGs (from Kim et al. 2020, n=832).

Supplementary Data 13. Shared RAGs in axotomized DRG at 1 dpi after PL conducted at ZT2-4 vs. unspecified ZT (Palmisano et al 2019) (n=815 genes). P value determined by Welch's twosample t-test.

Supplementary Data 14. qRT-PCR primers.
