# Supplementary material for: Circadian clock regulator Bmal1 gates axon regeneration via Tet3 epigenetics in mouse sensory neurons
Source: Nat Commun. 2023 Aug 24;14:5165. doi: 10.1038/s41467-023-40816-7 (PMC10449865; doi:10.1038/s41467-023-40816-7)
Supplement: Supplementary file 18 — Reporting Summary [file 41467_2023_40816_MOESM18_ESM.pdf]

nature portfolio

Corresponding author(s): Hongyan Zou

Last updated by author(s): Jul 29, 2023

Reporting Summary

Nature Portfolio wishes to improve the reproducibility of the work that we publish. This form provides structure for consistency and transparency in reporting. For further information on Nature Portfolio policies, see our Editorial Policies and the Editorial Policy Checklist.

Statistics

For all statistical analyses, confirm that the following items are present in the figure legend, table legend, main text, or Methods section.

n/a	Confirmed	
☐	☒	The exact sample size (n) for each experimental group/condition, given as a discrete number and unit of measurement
☐	☒	A statement on whether measurements were taken from distinct samples or whether the same sample was measured repeatedly
☐	☒	The statistical test(s) used AND whether they are one- or two-sided<br>*Only common tests should be described solely by name; describe more complex techniques in the Methods section.*
☒	☐	A description of all covariates tested
☐	☒	A description of any assumptions or corrections, such as tests of normality and adjustment for multiple comparisons
☐	☒	A full description of the statistical parameters including central tendency (e.g. means) or other basic estimates (e.g. regression coefficient) AND variation (e.g. standard deviation) or associated estimates of uncertainty (e.g. confidence intervals)
☐	☒	For null hypothesis testing, the test statistic (e.g. F, t, r) with confidence intervals, effect sizes, degrees of freedom and P value noted<br>*Give P values as exact values whenever suitable.*
☒	☐	For Bayesian analysis, information on the choice of priors and Markov chain Monte Carlo settings
☒	☐	For hierarchical and complex designs, identification of the appropriate level for tests and full reporting of outcomes
☒	☐	Estimates of effect sizes (e.g. Cohen's d, Pearson's r), indicating how they were calculated

Our web collection on statistics for biologists contains articles on many of the points above.

Software and code

Policy information about availability of computer code

Data collection	Zeiss AxioVision Rel 4.8 (fluorescent microscopy images); Zeiss Zen (Confocal images); Olympus cellSense Entry (Brightfield microscopy images); LI-COR Odyssey Classic system (western blotting); ABI Prism 7900HT Sequence Detection instrument (qRT-PCR);Illumina NovaSeq platform (RNA-seq).
Data analysis	Software used: GraphPad Prism 8.0 and Origin 2020b (plotting, statistical analysis); Fiji (image quantifications); Adobe Illustrator CS5 (illustration preparation); Microsoft Powerpoint and Microsoft Paint (figure preparation); Microsoft Excel (data handling); Ingenuity Pathway Analysis; GREAT 4.0 (http://great.stanford.edu/public/html/index.php); HOMER (Perl script findMotifsGenome.pl); deepTools software (Chromatin Accessibility Analysis); Eukaryotic Promoter Database platform (https://epd.epfl.ch//index.php); SDS 2.4 (qRT-PCR analysis); String dB (https://string-db.org/cgi/network.pl); NGS-Data-Charmer pipeline ; Trim-Galore tool (v0.6.5); Bowtie2(v2.4.1); SAMtools120 (v1.10)

For manuscripts utilizing custom algorithms or software that are central to the research but not yet described in published literature, software must be made available to editors and reviewers. We strongly encourage code deposition in a community repository (e.g. GitHub). See the Nature Portfolio guidelines for submitting code & software for further information.

Data

Policy information about availability of data

All manuscripts must include a data availability statement. This statement should provide the following information, where applicable:

- Accession codes, unique identifiers, or web links for publicly available datasets
- A description of any restrictions on data availability
- For clinical datasets or third party data, please ensure that the statement adheres to our policy

The RNA-seq data has been deposited at the NCBI GEO database under accession number GSE233367 [https://www.ncbi.nlm.nih.gov/geo/query/acc.cgi?acc=GSE233367]. Mouse genome mm9 and mm10. DRG 5hmC sequencing dataset17 is available under accession code: GSE85972. [https://www.ncbi.nlm.nih.gov/geo/query/acc.cgi?acc=GSE85972]. Published RNA-seq and ATAC-seq datasets14 are available at the GEO under accession codes: GSE97090 [https://www.ncbi.nlm.nih.gov/geo/query/acc.cgi?acc=GSE97090] and GSE132382 [https://0-www-ncbi-nlm-nih-gov.brum.beds.ac.uk/geo/query/acc.cgi?acc=GSE132382].

Human research participants

Policy information about studies involving human research participants and Sex and Gender in Research.

Reporting on sex and gender	Not applicable
Population characteristics	Not applicable
Recruitment	Not applicable
Ethics oversight	Not applicable

Note that full information on the approval of the study protocol must also be provided in the manuscript.

Field-specific reporting

Please select the one below that is the best fit for your research. If you are not sure, read the appropriate sections before making your selection.

☒ Life sciences ☐ Behavioural & social sciences ☐ Ecological, evolutionary & environmental sciences

For a reference copy of the document with all sections, see nature.com/documents/nr-reporting-summary-flat.pdf

Life sciences study design

All studies must disclose on these points even when the disclosure is negative.

Sample size	The number of mice, DRGs, and cells used for all experiments is indicated clearly in the figure legends and was determined based on previous experience and consistent with publications from our group and other labs the field (please see references below).<br>DOI: https://doi.org/10.1523/JNEUROSCI.5397-08.2009<br>DOI: 10.1073/pnas.1100426108<br>DOI: 10.1016/j.neuron.2015.09.050<br>DOI: 10.1016/j.neuron.2017.03.034<br>DOI: 10.1523/JNEUROSCI.0589-13.2013<br>In vivo analysis of nerve regeneration utilized 5 to 9 mice per group. In vitro DRG neuron culture and qRT-PCR analysis utilized the indicated number of DRGs obtained from a minimum of 3 mice per group unless otherwise indicted. N2a coimmunoprecipitation experiments were performed 3 independent times on different days. siRNA treatment of N2a and qRT-PCR analysis were performed on 2-and 5 different passages, respectively. qRT-PCR analyses of H9 stem cells, NPC, and cortical neurons, were performed in 3 independent conditions. Drug treatment of induced cortical neurons was performed on 3 independently derived cultures. DRG explants were obtained from 2 mice per group and at least 2 DRGs per mouse were used for analysis.
Data exclusions	No data were excluded from the analysis.
Replication	For all in vivo studies, individual animals were used as replicates. Experiments were repeated a minimum of 3 times and up to 6 times in different mice. In vitro experiments were replicated a minimum of 3 times as indicated. We observed that baseline 5hmC levels in DRG neurons can be affected by stress in mice caused by fighting or if analyzed immediately after 5 consecutive tamoxifen injections (once daily by IP). In the early of phase of the study, we optimized to wait at least 2 weeks after last injection before conducting experiments on Bmal1cKO mice. Mice with prior history of fighting were excluded from experiment. These observations are consistent with reported effects of stress and activity level on circadian clock (reference: doi: 10.1016/j.ynstr.2016.09.001) All other attempts at replication were successful.
Randomization	For all experimental groups, group allocation was randomized by age and sex. Littermates were assigned as controls and sex matched when possible.
Blinding	The investigators were not fully blinded during experiments and data collection, while data analyses were performed in a blinded manner

wherever possible, frequently involving two experimenters independently participating in quantifications.

Reporting for specific materials, systems and methods

We require information from authors about some types of materials, experimental systems and methods used in many studies. Here, indicate whether each material, system or method listed is relevant to your study. If you are not sure if a list item applies to your research, read the appropriate section before selecting a response.

Materials & experimental systems

n/a	Involved in the study
☐	☒ Antibodies
☐	☒ Eukaryotic cell lines
☒	☐ Palaeontology and archaeology
☐	☒ Animals and other organisms
☒	☐ Clinical data
☒	☐ Dual use research of concern

Methods

n/a	Involved in the study
☒	☐ ChIP-seq
☒	☐ Flow cytometry
☒	☐ MRI-based neuroimaging

Antibodies

Antibodies used

Primary antibodies used were as follows:
anti- Bmal1 (Novus, NB100-2288, 1:250),
TET3 (Active Motif, #61744, 1:300),
5hmC (Active Motif, #39769, 1:500),
pCREBS133(Cell Signaling, #9198, 1:300),
β-catenin (BD Biosciences, #610153, 1:200),
TUJ1/β3 tubulin (Biolegend, #801202, 1:1000; Cell Signaling, #5568, 1:300),
SCG10 (Novus, NBP1-49461, 1:1000),
CD206 (R&D systems, AF2535, 1:200),
CD68 (Bio-Rad, MCA1957GA, 1:200),
IBA1 (Wako, #019-19741, 1:2000),
ATF3 (Santa Cruz, sc-188, 1:300),
PGP9.5 (Neuromics, RA12103),
NFH (EMD Millipore, AB5539),
GFP (AVES, AB5541, 1:1000)
GAP43 (Millipore, AB5220, 1:300)
NPY (Santa Cruz, sc-133080, 1;100)
β-Actin (Sigma, A1978, 1:10,000)
mcherry (Invitrogen, M11240, 1:500)
Secondary cross-adsorbed and Alexa-coupled donkey anti-IgG antibodies (Jackson Immunoresearch) were used at a dilution of 1:300.

Validation

All antibodies used in this study have been validated by the companies from which they were purchased. Details about validation statements of the antibody manufacturers, relevant citations and antibody profiles can be found on the manufacturer's website.
anti- Bmal1 (Novus, NB100-2288, 1:250), https://www.novusbio.com/products/bmal1-antibody_nb100-2288

TET3 (Active Motif, #61744, 1:300), https://www.activemotif.com/catalog/details/61743/tet3-antibody-mab-clone-23b9

5hmC (Active Motif, #39769, 1:500), https://www.activemotif.com/catalog/details/39769.html

pCREBS133(Cell Signaling, #9198, 1:300), https://www.cellsignal.com/products/primary-antibodies/phospho-creb-ser133-87g3-rabbit-mab/9198

β-catenin (BD Biosciences, #610153, 1:200), https://www.bdbiosciences.com/en-us/products/reagents/microscopy-imaging-reagents/immunofluorescence-reagents/purified-mouse-anti-catenin.610153

TUJ1/β3 tubulin (Biolegend, #801202, 1:1000; Cell Signaling, #5568, 1:300), https://www.biolegend.com/fr-fr/products/purified-anti-tubulin-beta-3-tubb3-antibody-11580?GroupID=GROUP686 and https://www.cellsignal.com/products/primary-antibodies/b3-tubulin-d71g9-xp-rabbit-mab/5568

SCG10 (Novus, NBP1-49461, 1:1000) https://www.novusbio.com/products/stathmin-2-stmn2-antibody_nbp1-49461

CD206 (R&D systems, AF2535, 1:200), https://www.bio-techne.com/p/antibodies/mouse-mmr-cd206-antibody_af2535

CD68 (Bio-Rad, MCA1957GA, 1:200), https://www.scbt.com/p/cd68-antibody-kp1?
gclid=CjwKCAjw8ZKmBhArEiwAspcJ7s1PchRm8dWX5dXjo3nqdKUqPvVlIe2MYziquwwux3HE0rL1V5m3nRoCwZQQAvD_BwE

IBA1 (Wako, #019-19741, 1:2000), https://labchem-wako.fujifilm.com/us/product/detail/W01W0101-1974.html

ATF3 (Santa Cruz, sc-188, 1:300), https://www.scbt.com/p/atf-3-antibody-c-19

PGP9.5 (Neuromics, RA12103), https://www.neuromics.com/RA12103

NFH (EMD Millipore, AB5539), https://www.emdmillipore.com/US/en/product/Anti-Neurofilament-H-Antibody,MM_NF-AB5539?ReferrerURL=https%3A%2F%2Fwww.google.com%2F&bd=1

GFP (AVES, AB5541, 1:1000) https://www.scbt.com/p/gfp-antibody-b-2?gclid=CjwKCAjw8ZKmBhArEiwAspcJ7k-CdaFl7w2FnykEb86IiZ65lpVmtDwmvvCs9fl66ZtvS0h2MOQZjhoCZ7MQAvD_BwE

GAP43 (Millipore, AB5220, 1:300) https://www.emdmillipore.com/US/en/product/Anti-Growth-Associated-Protein-43-GAP-43-Antibody,MM_NF-AB5220?ReferrerURL=https%3A%2F%2Fwww.google.com%2F

NPY (Santa Cruz, sc-133080, 1;100) https://www.scbt.com/p/npy-antibody-f-6

β-Actin (Sigma, A1978, 1:10,000) https://www.sigmaaldrich.com/US/en/product/sigma/a1978

mcherry (Invitrogen, M11240, 1:500) https://www.thermofisher.com/antibody/product/mCherry-Antibody-clone-16D7-Monoclonal/M11240

Eukaryotic cell lines

Policy information about cell lines and Sex and Gender in Research

Cell line source(s)	Neuro2a ATCC CCL-131, H9 hESC (StemCellCore@mssm.edu)
Authentication	Cell-lines were obtained directly from source and were not further validated in the lab.
Mycoplasma contamination	Cell lines was tested negative for mycoplasma contamination by using IDEXX RADIL service.
Commonly misidentified lines (See ICLAC register)	No commonly misidentified cell lines were used in this work.

Animals and other research organisms

Policy information about studies involving animals; ARRIVE guidelines recommended for reporting animal research, and Sex and Gender in Research

Laboratory animals	Mouse strains were obtained from The Jackson Laboratory (C57BL/6J-stock #000664), Tg(Thy1-cre/ERT2,-EYFP)HGfng (also known as SLICK-H-stock #012708), B6.Cg-Tg(Nes-cre)1Kln/J (stock #003771) , Bmalfl/fl (stock #007668); or Charles River CD-1 mice. Animal procedures were conducted according to a protocol (IPROTO202200000184) approved by the Institutional Animal Care and Use Committee (IACUC) at Mount Sinai. All mice were bred onto C57BL/6J genetic background for at least three generations. Animals were housed under 12:12 hr light:dark cycle with ad libitum access to food and water and were habituated to the facility for at least 2 weeks prior to conducting experiments. Facility temperature is estimated at 18-23°C with 40-60% humidity. Animals were housed in groups of 5 in pathogen free barrier facility, in corn bedding lined cages, with pellet chow and water bottles. All mice used in the study were used as young adults of less than 16 week of age.
Wild animals	The study did not involve wild animals.
Reporting on sex	We used mixed sex for all experiments. We considered the effect of sex in Figure 9C and found no apparent effect on neurite outgrowth of DRG neurons.
Field-collected samples	This study does not involve field work.
Ethics oversight	All animal procedures complied with ethical regulations for animal testing and research and were performed according to protocols approved by the Institutional Animal Care and Use Committee (IACUC) at Icahn School of Medicine at Mount Sinai (approved protocol# IPROTO202200000184). H9 human embryonic stem (hES) cells studies were approved by the Embryonic Stem Cell Research Oversight Committee (ESCRO) at Icahn School of Medicine at Mount Sinai.

Note that full information on the approval of the study protocol must also be provided in the manuscript.
